# Altered albumin/neutrophil to lymphocyte ratio are associated with all-cause and cardiovascular mortality for advanced cardiovascular-kidney-metabolic syndrome

**DOI:** 10.3389/fnut.2025.1595119

**Published:** 2025-07-16

**Authors:** Xiaoshuang Yin, Jinmei Zou, Jing Yang

**Affiliations:** Department of Immunology, Mianyang Central Hospital, School of Medicine, University of Electronic Science and Technology of China (UESTC), Mianyang, China

**Keywords:** ANLR, malnutrition, inflammation, mortality, advanced CKM syndrome, NHANES

## Abstract

**Background:**

Advanced cardiovascular-kidney-metabolic (CKM) syndrome refers to stages 3 and 4 of CKM syndrome, which are associated with higher mortality compared to earlier stages (0–2). The albumin (ALB)-to-neutrophil/lymphocyte ratio (ANLR) is a new predictive marker that participates in immune inflammation and dietary status. However, the influence of ANLR on all-cause mortality (ACM) and cardiovascular mortality (CVM) in individuals with advanced CKM syndrome remains unclear. This investigation aims to examine the link between ANLR and both ACM and CVM in this population using data from a large-scale cross-sectional survey in the United States.

**Methods:**

Data were from the National Health and Nutrition Examination Survey (NHANES) spanning 1999 to 2018, a nationally representative cross-sectional survey with longitudinal mortality follow-up from the National Death Index. The formula of ANLR is ALB/NLR. The diagnostic criteria of CKM syndrome was based on the concept proposed by the American Heart Association and modified criteria adapted for NHANES data availability. The outcomes of interested included ACM and CVM. A 1:1 propensity score matching (PSM) approach was used to control for potential confounding variables. The threshold value of ANLR influencing survival was determined using maximally selected rank statistics, which is based on the log-rank test. This method identifies the optimal cutoff for continuous variables where the difference in survival rates is most pronounced, making it particularly well-suited for analyzing time-to-event data, such as survival outcomes. Kaplan–Meier survival analysis and multivariate Cox proportional hazards models were employed to assess the effects of ANLR on both ACM and CVM. Restricted cubic spline (RCS) analysis evaluated the linear or non-linear association between ANLR and mortality outcomes. Stratified analysis and interaction testing were carried out to estimate the influence of covariates on the ANLR-mortality correlation.

**Results:**

A total of 3,266 adults with advanced CKM syndrome (41.12% male) were included in the analysis, with median (interquartile range) age of 73 (63–80). Prior to PSM, and fully adjustment, the lowest ANLR Tertile 1 was related to significantly higher risks of ACM (hazard ratio [HR]: 1.58, 95% confidence interval [CI]: 1.39–1.78, *p* < 0.001) and CVM (HR: 1.65, 95% CI: 1.34–2.04, *p* < 0.001) compared to the highest Tertile 3. After applying PSM, and fully adjusting for confounders, an ANLR score below 1.04 was independently linked to increased risks of both CVM (HR: 2.02, 95% CI: 1.49–2.75, *p* < 0.001) and ACM (HR: 1.52, 95% CI: 1.27–1.81, *p* < 0.001). Interaction tests revealed no significant interactions for CVM across subgroups (All *P*_interaction_ > 0.05). Regarding ACM, interactions were noted between ANLR and age, gender, and CKM stages (All *P*_interaction_ < 0.05). RCS analysis indicated an L-shaped link between ANLR and both ACM and CVM, both before and after PSM (all *P*_non-linearity_ < 0.001). The predictive value of ANLR, NLR, and ALB for CVM and ACM in individuals with advanced CKM syndrome demonstrated that ANLR and NLR exhibited comparable predictive capabilities for both ACM and CVM, outperforming ALB. Furthermore, the predictive performance of ANLR and NLR for ACM was superior to that for CVM.

**Conclusion:**

Lower ANLR values, indicative of elevated systemic inflammation and malnutrition, are independently linked to increased risks of both ACM and CVM in individuals with advanced CKM syndrome in the US. These readily accessible and low-cost blood markers could serve as valuable prognostic indicators for identifying high-risk individuals. Future research should focus on incorporating additional biomarkers, validating the indices in larger and more diverse cohorts, and employing advanced analytical methods to refine the diagnostic efficiency of ANLR and NLR for better clinical utility.

## Introduction

In October 2023, the American Heart Association (AHA) proposed the concept of cardiovascular-kidney-metabolic (CKM) syndrome, highlighting the interconnection between metabolic disorders, including diabetes and obesity, and both renal and cardiovascular disorders (1). CKM syndrome is identified as a systemic condition recognized by complex interactions among metabolic risk factors, chronic kidney disease (CKD), and cardiovascular diseases (CVDs) (1). This hypothesis is progressively substantiated by scientific research, illustrating a constant association among metabolic dysfunctions, CVDs, and CKD, with these illnesses often co-occurring in patients (2, 3). All of these diseases are associated with reduced survival rates and increased mortality risks (3). The common pathogenic pathways indicate that the advancement of one disease may aggravate the others, potentially resulting in deteriorated clinical outcomes for those affected. Advanced CKM syndrome corresponds to stages 3 and 4 of CKM syndrome (4). Individuals in these stages face a very high risk of CKD and a 10-year risk of CVDs. Many also have pre-existing CVDs, such as coronary heart disease (CHD), angina, myocardial infarction (MI), heart failure (HF), or stroke (4). Compared to those with non-advanced CKM syndrome (stages 0–2), individuals in the advanced stages experience higher mortality. This ongoing burden presents a significant public health threat, emphasizing the requirement for continued attention to approaches of prevention and treatment targeting modifiable risk factors to reduce its global impact.

Emerging literature further supports the interconnectedness of cardiovascular and renal dysfunction. Zhao et al. (5) proposed a classification of cardiorenal syndromes and emphasized shared mechanisms such as hemodynamic imbalance, neurohormonal activation, oxidative stress, inflammation, and fibrosis. Zhang et al. (6) identified trimethylamine oxide, a gut microbial metabolite, as a key circulating mediator implicated in both heart failure and CKD progression, highlighting the gut–heart–kidney axis. In parallel, Zhu et al. (7) summarized how clinical features of CKD–CVD often diverge from typical CVD presentations, with nontraditional factors (e.g., uremic toxins, mineral metabolism disorder) playing key roles. They also advocated for integrated Chinese and Western medicine approaches to delay progression. Additionally, a recent review (8) highlighted the utility of biomarkers like NT-proBNP and Galectin-3 for early recognition of cardiorenal dysfunction and emphasized proactive intervention strategies.

Systemic inflammation has a central function in several chronic disorders, influencing the cardiovascular, renal, and metabolic systems (9–11). It is characterized by increased concentrations of pro-inflammatory cytokines such as TNF-*α*, IL-6, and C-reactive protein, which facilitate atherosclerosis, endothelial dysfunction, and vascular rigidity, contributing to hypertension and HF (12, 13). In renal disorders, inflammation damages the kidneys through glomerular damage and fibrosis, accelerating CKD progression (14). Inflammation is metabolically related to insulin resistance, a hallmark of type 2 diabetes and metabolic syndrome, which exacerbates visceral fat accumulation and subsequent inflammation (15).

The neutrophil-to-lymphocyte ratio (NLR), measured from neutrophil and lymphocyte numbers, offers a measurement of systemic inflammation, with higher NLR indicating greater inflammation. Investigations have established NLR as a reliable inflammation marker, illustrating its prognostic value in oncology, diabetes, and CVDs (16–19), where elevated NLR levels are associated with worse outcomes (20–23). Additionally, albumin (ALB), frequently employed to assess dietary status, has a strong association with the occurrence and death risk of several chronic disorders (24–28).

The ALB / neutrophil-to-lymphocyte ratio (ANLR), which combines the neutrophil-to-lymphocyte ratio (NLR) and ALB, has appeared as a novel integrated index for assessing both inflammation and nutritional status. Recent studies have indicated that ANLR is negatively correlated with diabetic foot ulcers (29). However, its relationship with mortality outcomes in advanced CKM syndrome remains to be investigated. We hypothesize that ANLR, due to its accessibility and relevance, is a suitable marker for exploring the interaction between nutrition, inflammation, and death in individuals with advanced CKM syndrome. This cross-sectional investigation represents the first attempt to evaluate the correlation between ANLR and mortality outcomes in this population using the National Health and Nutrition Examination Survey (NHANES) data. Our findings aim to provide valuable insights for developing personalized strategies to prevent mortality in advanced CKM syndrome.

## Methods

### Study design and participants

The NHANES is a set of cross-sectional surveys with a complicated sampling strategy intended to assess the health and dietary status of the non-institutionalized US population across all age groups (30). Each participant was monitored until death. This cross-sectional cohort investigation included outcomes from the 1999–2018 NHANES cycles.

**Figure 1:**
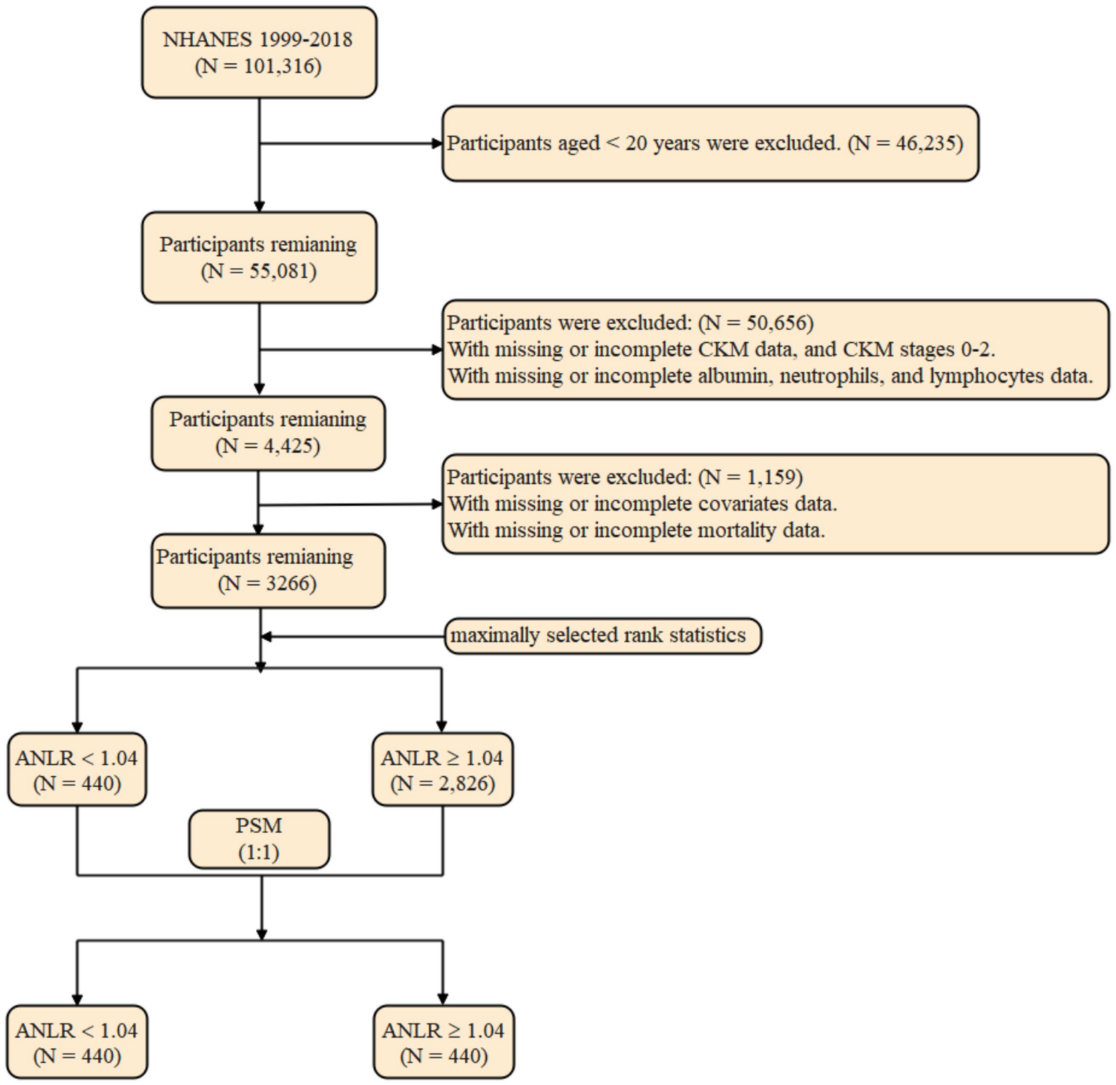
Participant selection algorithm for the NHANES 1999–2018 analytic cohort.

The initial sample included 101,316 participants from the NHANES fasting subsample (1999–2018). After excluding 40,235 individuals aged under 20 years, 55,081 participants remained. Additional exclusions were made for those with insufficient data for diagnosing CKM syndrome (n = 29,858), those diagnosed with CKM syndrome stages 0–2 (*n* = 20,547), and participants missing data on ALB, neutrophil, or lymphocyte counts (*n* = 251). This left 4,425 participants for analysis. Additional exclusions were implemented due to absent covariate (*n* = 1,158) and mortality data (*n* = 1). The final cohort for analysis comprised 3,266 participants (Figure 1).

The NHANES protocol received approval from the Institutional Review Board for the National Center for Health Statistics (currently known as the Ethics Review Board), which permitted the release of the data files for public use. A written informed consent was provided from each participant prior to data collection. This study adhered to the Strengthening the Reporting of Observational Studies in Epidemiology (STROBE) instructions for cohort investigations.

### ANLR calculation, and determination of optimal threshold value of ANLR

Data on ALB, neutrophil, and lymphocyte counts were collected, and the ANLR was computed using the formula previously outlined (29):


$$\text{ANLR}=\frac{\mathrm{ALB}\;(g/\mathrm{dL})}{\mathrm{NLR}};\mathrm{NLR}=\frac{\text{Neutrophil}\;(\times 10^{9}/L)}{\text{Lymphocyte}\;(\times 10^{9}/L)}$$

**Figure 2:**
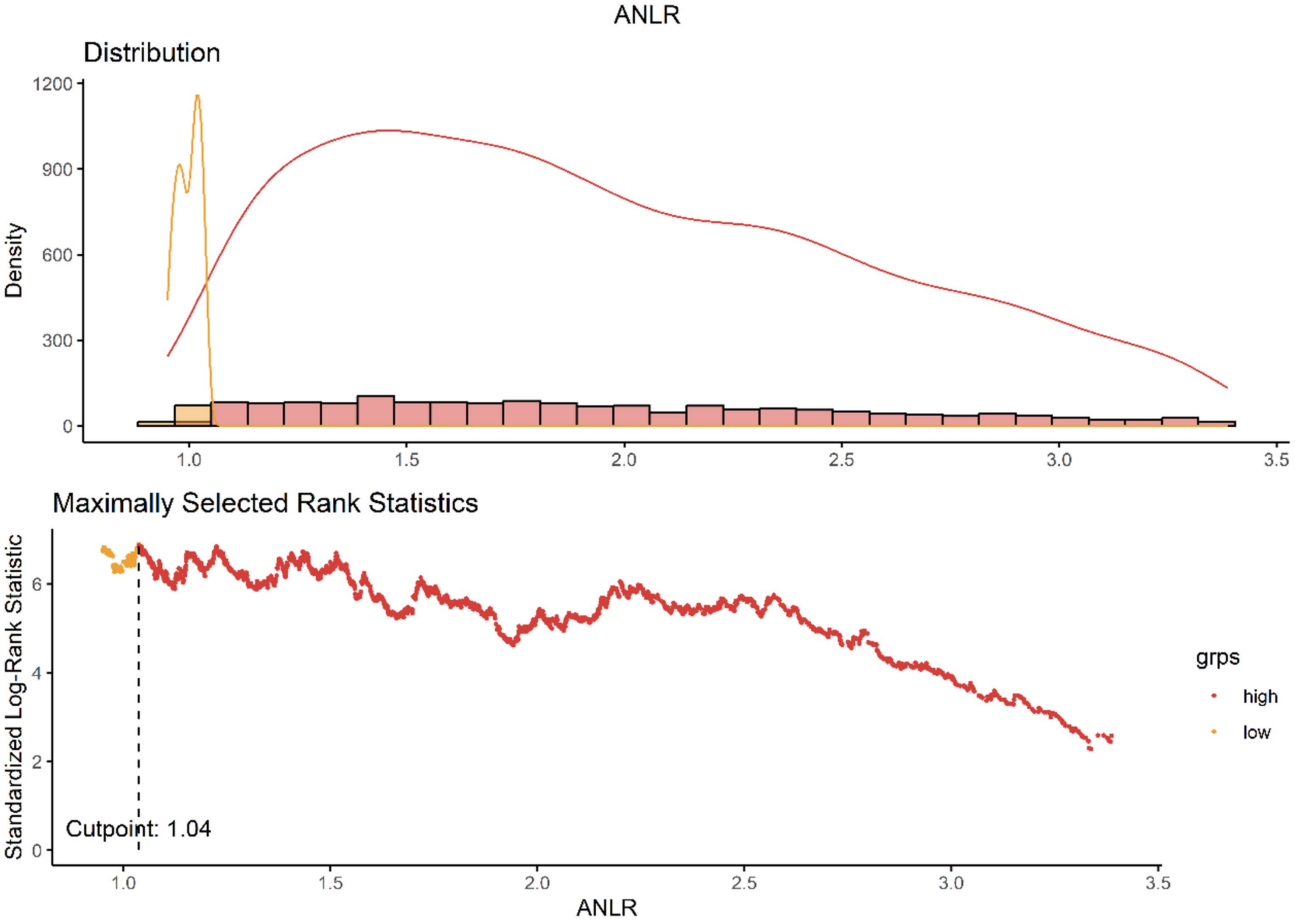
ANLR cutoff determination using maximized rank statistics.

The ANLR were computed using values obtained from liver function tests and routine blood tests. Currently, no established cutoffs exist to differentiate high from low values for ANLR. To address this, we employed the maximally selected rank statistics method to identify the optimal threshold points for it (31). This statistical technique, which is based on the log-rank test, identifies the most significant cutoff in continuous variables where the disparity in survival rates is most pronounced. It is particularly well-suited for time-to-event data, such as survival outcomes. Using this data-driven approach, the identified cutoff values were 1.04 for ANLR (Figure 2).

To mitigate the risk of overfitting, we applied a Bonferroni-adjusted significance level and further performed internal validation via bootstrap resampling (*n* = 1,000). The Bootstrap analysis indicates that the distribution of the cutoff values of ANLR is concentrated and close to 1.04, suggesting that the result is stable. This stability reflects the minimal fluctuation of the cutoff values during repeated sampling, further validating the reliability of the model estimation. Moreover, the 95% confidence interval (CI) is ranging from 0.942 to 2.497, and the original cutoff value of 1.04 falls within this interval. This indicates that the cutoff estimate is statistically reliable. Therefore, considering both the Bootstrap distribution and the CI, the original cutoff estimate can be regarded as stable and robust (Supplementary Figure S1). Figure 2 illustrates the distribution of ANLR values and their relationship with mortality outcomes, highlighting the association between ANLR values < 1.04 and increased mortality risk. Following this, participants were stratified into two distinct groups based on the threshold value.

### Assessment of CKM syndrome

CKM syndrome was classified into five stages (0–4) based on modified criteria adapted for NHANES data availability, referencing the AHA definitions (1) and prior large-scale analyses (30, 32). Due to the absence of certain diagnostic tools (e.g., coronary artery calcium [CAC] scores, NT-proBNP, echocardiography, Ankle-Brachial Index [ABI]), we used proxy indicators as detailed below.

Stage 0: Participants with normal body mass index (BMI) (<25 kg/m^2^), waist circumference (WC) (< 88 cm for women, < 102 cm for men), and these participants will not be classified into a higher stage category (normoglycemia, normotension, normal lipid profile, no evidence of CKD or clinical/subclinical CVD).

Stage 1: Participants with elevated BMI (≥ 25 kg/m^2^), increased waist circumference (≥ 88 cm for women, ≥ 102 cm for men), or prediabetes (hemoglobin A1c [HbA1c]: 5.7–6.4% or fasting blood glucose [FBG]: 100–125 mg/dL), without the presence of other metabolic risk factors or CKD.

Stage 2: Participants with metabolic risk factors or moderate-to-high-risk CKD per KDIGO guidelines (33). Metabolic risk factors included elevated triglycerides (TG) (≥ 135 mg/dL), hypertension, diabetes, or metabolic syndrome (≥ 3 of the following: elevated WC, low HDL [< 40 mg/dL for men, < 50 mg/dL for women], elevated TG [≥150 mg/dL], elevated blood pressure [systolic ≥130 mmHg, diastolic ≥80 mmHg], or prediabetes).

Stage 3: Participants with very high CKD risk was characterized by either Stage G4 or G5 CKD (eGFR < 30 mL/min/1.73 m^2^) or a classification of very high risk based on KDIGO guidelines, determined by GFR and UACR (eGFR < 30 mL/min/1.73m^2^ or UACR ≥ 300 mg/g) (33), or a calculated 10-year atherosclerotic cardiovascular disease risk (ASCVD) ≥ 20% [https://professional.heart.org/en/guidelines-and-statements/prevent-calculator], based on pooled cohort equations.

Stage 4: Participants with a self-reported physician diagnosis of CHD, stroke, angina, CHF, or MI. Atrial fibrillation (AF) and peripheral artery disease (PAD) were not included due to data unavailability.

Advanced CKM syndrome was defined as encompassing stages 3 and 4 of CKM syndrome, in alignment with the classification by Zhu et al. (4). The specific NHANES variables used for each criterion are listed in Supplementary Table S1.

### Outcomes

This study evaluated two mortality outcomes: all-cause mortality (ACM) and cardiovascular mortality (CVM). ACM refers to death from any cause, while CVM specifically pertains to death attributed to CVDs. Mortality outcomes were acquired from the National Death Index (NDI) and linked to NHANES data up to December 31, 2019.

### Covariates

Drawing from existing literature and biological plausibility, a comprehensive set of covariates known to influence outcomes in advanced CKM syndrome was considered. These included demographic factors (age, sex, and race), socioeconomic predictors (marital status, educational attainment, and the family poverty-income ratio [PIR]), lifestyle and health habits (BMI, smoking habits, alcohol intake), and comorbidities such as hypertension, diabetes, hyperlipidemia, liver condition, CHD, CHF, MI, angina, stroke, CKD, and cancer. Laboratory parameters, including neutrophil, lymphocyte, ALB, TG, high-density lipoprotein (HDL), and HbA1c, were also included. By adjusting for these factors, we sought to minimize confounding influences and enhance the robustness of our analysis on the relationship between ANLR and both CVM and ACM in individuals with advanced CKM syndrome.

### Propensity score matching

Due to the retrospective design of this study, which is susceptible to selection bias and possible confounding variables, propensity score matching (PSM) was utilized to rectify these concerns and reduce their influence. A logistic regression model was utilized to produce propensity scores, and individuals were thereafter matched in a 1:1 ratio according to these scores. The covariates’ selection for the PSM procedure was informed by an extensive review of relevant literature. These covariates included demographic factors, socioeconomic indicators, lifestyle factors, clinical characteristics, and laboratory parameters. This rigorous selection process ensured the inclusion of scientifically relevant and contextually appropriate covariates.

To minimize discrepancies between matched pairs, the closest neighbor matching algorithm was applied with a caliper width of 0.1. The effectiveness of the PSM in balancing baseline characteristics between groups was evaluated by calculating the absolute standardized differences (ASDs). ASD values below 0.10 following matching indicated that the potential biases and confounding factors had been effectively addressed, confirming a fair comparison between the groups for subsequent analyses.

### Statistical analysis

We applied methods to account for masked variance and used a weighting approach (34). Continuous variables were expressed as means with interquartile ranges (IQR) and compared via the t-test or Mann–Whitney U-test based on normality evaluated by the Shapiro–Wilk test. Categorical variables were reported as counts and proportions, with comparisons made using Fisher’s exact test or the Chi-square test. The ideal ANLR cutoff for predicting ACM and CVM was determined using maximally selected rank statistics, set at 1.04 to optimize the risk ratio.

To validate the proportional hazards (PH) assumption, we utilized both graphical and statistical methods. The statistical assessment was performed using Schoenfeld residuals and the Grambsch-Therneau test, while visual validation was carried out via Kaplan–Meier (KM) survival curves. The Cox PH model was used to account for censored data, considering individuals who had no experience of the event during the investigation as non-events. The analysis followed each participant’s timeline from the baseline assessment to either death or the study endpoint.

The Cox PH model identified significant predictors of mortality through both univariate and multivariate analyses. Outcomes are presented as hazard ratios (HRs) with 95% confidence intervals (CIs). Model 1 was unadjusted; Model 2 was adjusted for age, sex, and race; Model 3 involved additional adjustments for education level, family PIR, tobacco consumption, alcohol intake, and BMI. Disease variables that are integral to CKM syndrome diagnosis—such as CHD, stroke, CKD, diabetes, hypertension, and hyperlipidemia—were excluded to avoid overadjustment and collider bias. Subgroup analyses were carried out to explore ANLR influence on mortality outcomes across different participant groups and comorbid conditions, using a multivariate Cox PH model. Factors considered included age (categorized into 20–59 years and ≥ 60 years), gender, race/ethnicity, hypertension, diabetes, hyperlipidemia, stroke, and CKM stages. Additionally, ANLR was divided into tertiles to examine its relationship with ACM and CVM, with comparisons made between the highest tertile (reference) and other groups.

Restricted cubic splines (RCS) were employed for adaptable curve fitting to examine possible non-linear associations. Generalized additive models were utilized to evaluate the impact of ANLR on mortality. The placement and quantity of knots were established based on previous research and preliminary analyses, specifically in the 10th, 50th, and 90th percentiles of the ANLR distribution, which were corroborated through first-model diagnostics. Statistical significance was deemed for two-sided testing at *p* < 0.05. Data analysis was conducted utilizing SPSS Statistics 26 and R statistical software (version 4.2.2) to guarantee thorough assessment.

## Results

### Characteristics of the included advanced CKM syndrome participants pre-PSM

The baseline characteristics and findings of individuals prior to PSM are summarized in Table 1. The total sample size was 3,266, with participants classified into two groups according to the ideal ANLR cutoff: low ANLR (440 participants) and high ANLR (2,826 participants). Internal validation via 1,000 bootstrap iterations yielded a median optimal cutoff of 1.04, suggesting that the data-driven threshold is relatively stable. The frequency distribution of bootstrap-derived cutoffs is shown in Supplementary Figure S1. The low ANLR group had a significantly higher median age (76.5 *vs.* 72.0 years) and a lower proportion of males (37.05% *vs.* 41.26%) compared to the high ANLR group. Significant variations were noticed between the groups in age, ethnicity, marital status, tobacco consumption, hyperlipidemia, stroke, CKD, CKM stage, and cancer, while no significant differences were found for gender, education level, family PIR, alcohol consumption, hypertension, diabetes, MI, angina, liver conditions, and BMI. Physiological markers indicated a significant elevation in neutrophil concentrations in the low ANLR group, while the high ANLR group demonstrated higher levels of ALB, lymphocytes, and TG. No significant differences were noted in HDL or HbA1c levels. Clinically, the low ANLR group exhibited significantly higher rates of CVM (26.14% vs. 17.34%, *p* < 0.001) and ACM (68.41% vs. 47.17%, p < 0.001) compared to the high ANLR group.

**Table 1:** Baseline characteristics and findings prior to PSM.

Variables	Overall (*n* = 3,266)	ANLR		*P-*value
		Low (*n* = 440)	High (*n* = 2,826)	
ANLR, median (IQR)	1.81 (1.31–2.49)	0.84 (0.69–0.95)	1.95 (1.51–2.64)	<0.001
Age, years, median (IQR)	73 (63–80)	76.5 (70–80)	72 (62–80)	<0.001
Sex, male, *n* (%)	1,343 (41.12)	163 (37.05)	1,180 (41.76)	0.06
Race, *n* (%)				<0.001
Mexican American	385 (11.79)	38 (8.64)	347 (12.28)	
Non-Hispanic Black	624 (19.11)	42 (9.54)	582 (20.59)	
Non-Hispanic White	1918 (58.73)	328 (74.55)	1,590 (56.26)	
Other Hispanic	198 (6.06)	18 (4.09)	180 (6.37)	
Other	141 (4.32)	14 (3.18)	127 (4.49)	
Marital status, *n* (%)				0.04
Divorced/Widowed/separated	1,223 (37.45)	188 (42.73)	1,035 (36.62)	
Married/Partnered	1894 (57.99)	236 (53.64)	1,658 (58.67)	
Never married	149 (4.56)	16 (3.64)	133 (4.71)	
Education, *n* (%)				0.93
Above HS	1,314 (40.23)	179 (40.68)	1,135 (40.16)	
HS or Equivalent	783 (23.97)	107 (24.32)	676 (23.92)	
Below HS	1,169 (35.79)	154 (35.00)	1,015 (35.92)	
Family PIR,%, *n* (%)				0.05
<1.30	1,087 (33.28)	128 (29.09)	959 (33.93)	
1.30–3.49	1,428 (43.72)	215 (48.86)	1,213 (42.92)	
≥3.50	751 (22.99)	97 (22.05)	654 (23.14)	
Smoking status, *n* (%)				0.01
Never	1,332 (40.78)	158 (35.91)	1,174 (41.54)	
Former	1,325 (40.57)	207 (47.05)	1,118 (39.56)	
Now	609 (18.65)	75 (17.05)	534 (18.90)	
Alcohol consumption, *n* (%)				0.11
Never	524 (16.04)	61 (13.86)	463 (16.38)	
Former	1,048 (32.09)	161 (36.59)	887 (31.39)	
Mild	1,177 (36.04)	160 (36.36)	1,017 (35.99)	
Moderate	247 (7.56)	25 (5.68)	222 (7.86)	
Heavy	270 (8.27)	33 (7.50)	237 (8.39)	
Hypertension, *n* (%)	2,534 (77.59)	354 (80.45)	2,180 (77.14)	0.12
Diabetes, *n* (%)	1,439 (44.06)	212 (48.18)	1,227 (43.42)	0.06
Hyperlipidemia, *n* (%)	2,758 (84.45)	344 (78.18)	2,414 (85.42)	<0.001
Stroke, *n* (%)	675 (20.67)	75 (17.05)	600 (21.23)	0.04
CHD, *n* (%)	755 (23.1)	115 (26.1)	640 (22.6)	0.11
CHF, *n* (%)	559 (17.1)	106 (24.1)	453 (16.0)	<0.001
MI, *n* (%)	772 (23.64)	119 (27.05)	653 (23.11)	0.07
Angina, *n* (%)	519 (15.89)	72 (16.36)	447 (15.82)	0.77
Liver condition, *n* (%)	185 (5.66)	31 (7.04)	154 (5.45)	0.18
CKD, *n* (%)	1,521 (46.57)	266 (60.45)	1,255 (44.41)	<0.001
CKM stages, *n* (%)				0.04
3	1,133 (34.69)	172 (39.09)	961 (34.01)	
4	2,133 (65.31)	268 (60.91)	1865 (65.99)	
Cancer, *n* (%)	692 (21.19)	139 (31.59)	553 (19.57)	< 0.001
Data presented as median (IQR)				
BMI (kg/m^2^)	28.2 (24.89–32.25)	27.6 (24.21–32.29)	28.28 (24.97–32.23)	0.07
ALB (g/dL)	4.1 (3.9–4.4)	4 (3.8–4.2)	4.2 (4–4.4)	< 0.001
Neutrophils (10^9^/L)	4.1 (3.2–5.2)	5.8 (4.8–6.9)	3.9 (3.1–4.8)	< 0.001
Lymphocytes (10^9^/L)	1.8 (1.4–2.3)	1.2 (0.9–1.5)	1.9 (1.5–2.3)	< 0.001
TG (mg/dL)	122 (86–176)	112 (80–165.5)	123 (87–178)	0.004
HDL-C (mg/dL)	48 (41–59)	47 (41–58)	48 (41–59)	0.32
HbA1c (%)	5.8 (5.4–6.4)	5.8 (5.4–6.5)	5.8 (5.4–6.4)	0.57
CVM, *n* (%)	605 (18.52)	115 (26.14)	490 (17.34)	<0.001
ACM, *n* (%)	1,634 (50.03)	301 (68.41)	1,333 (47.17)	<0.001

ANLR: albumin/neutrophil to lymphocyte ratio; HS: high school; PIR: poverty-income ratio; CHD: coronary heart disease; CHF: chronic heart failure; MI: myocardial infarction; CKD: chronic kidney disease; CKM: cardiovascular-kidney-metabolic syndrome; BMI: body mass index; ALB: albumin; TG: triglycerides; HDL-C: high-density lipoprotein; hemoglobin A1c: HbA1c; CVM: cardiovascular mortality; ACM: all-cause mortality.

### Associations of the ANLR with mortality outcomes in advanced CKM syndrome participants pre-PSM

Table 2 presents the outcomes of both univariate and multivariate Cox PH analyses for CVM and ACM before PSM across three models of adjustment. Regarding CVM, participants with an ANLR score < 1.04 exhibited a significantly raised risk compared to those with higher ANLR scores, with HRs of 2.23 (Model 1), 1.80 (Model 2), and 1.81 (Model 3), all with *p*-values < 0.001. Analysis by tertiles further demonstrated that the lowest tertile (T1) consistently showed a significantly higher risk than the highest tertile (T3), with HRs of 2.06, 1.66, and 1.65 across the three models (all *p* < 0.001). For ACM, an ANLR score < 1.04 was also associated with increased risk, with HRs of 2.14 (Model 1), 1.75 (Model 2), and 1.77 (Model 3), all *p* < 0.001. The T1 group exhibited HRs of 1.94, 1.58, and 1.58 across the three models, all with *p*-values < 0.001.

**Table 2:** HR (95% CIs) for connection between ANLR and mortality outcomes in advanced CKM syndrome pre-PSM.

Outcomes	Models					
	I		II		III	
	HR (95% CI)	*p*-value	HR (95% CI)	*p*-value	HR (95% CI)	*p*-value
CVM						
ANLR (<1.04)	2.23 (1.82–2.74)	<0.001	1.80 (1.46–2.21)	<0.001	1.81 (1.47–2.23)	<0.001
ANLR (tertiles)						
T1	2.06 (1.68–2.52)	<0.001	1.66 (1.34–2.04)	<0.001	1.65 (1.34–2.04)	<0.001
T2	1.41 (1.15–1.74)	0.001	1.27 (1.03–1.56)	0.03	1.25 (1.01–1.54)	0.04
T3	*Ref.*		*Ref.*		*Ref.*	
*P* for trend		<0.001		<0.001		<0.001
ACM						
ANLR (<1.04)	2.14 (1.89–2.43)	<0.001	1.75 (1.54–1.99)	<0.001	1.77 (1.56–2.01)	<0.001
ANLR (tertiles)						
T1	1.94 (1.71–2.18)	<0.001	1.58 (1.39–1.79)	<0.001	1.58 (1.39–1.78)	<0.001
T2	1.29 (1.14–1.47)	<0.001	1.17 (1.03–1.33)	0.02	1.15 (1.01–1.30)	0.04
T3	*Ref.*		*Ref.*		*Ref.*	
*P* for trend		< 0.001		< 0.001		< 0.001
Model 1 was unadjusted;						
Model 2 was adjusted for age, sex, and race;						
Model 3 involved additional adjustments for education level, family PIR, tobacco consumption, alcohol intake, and BMI.						

ANLR: albumin/neutrophil to lymphocyte ratio; CVM: cardiovascular mortality; ACM: all-cause mortality.

**Figure 3:**
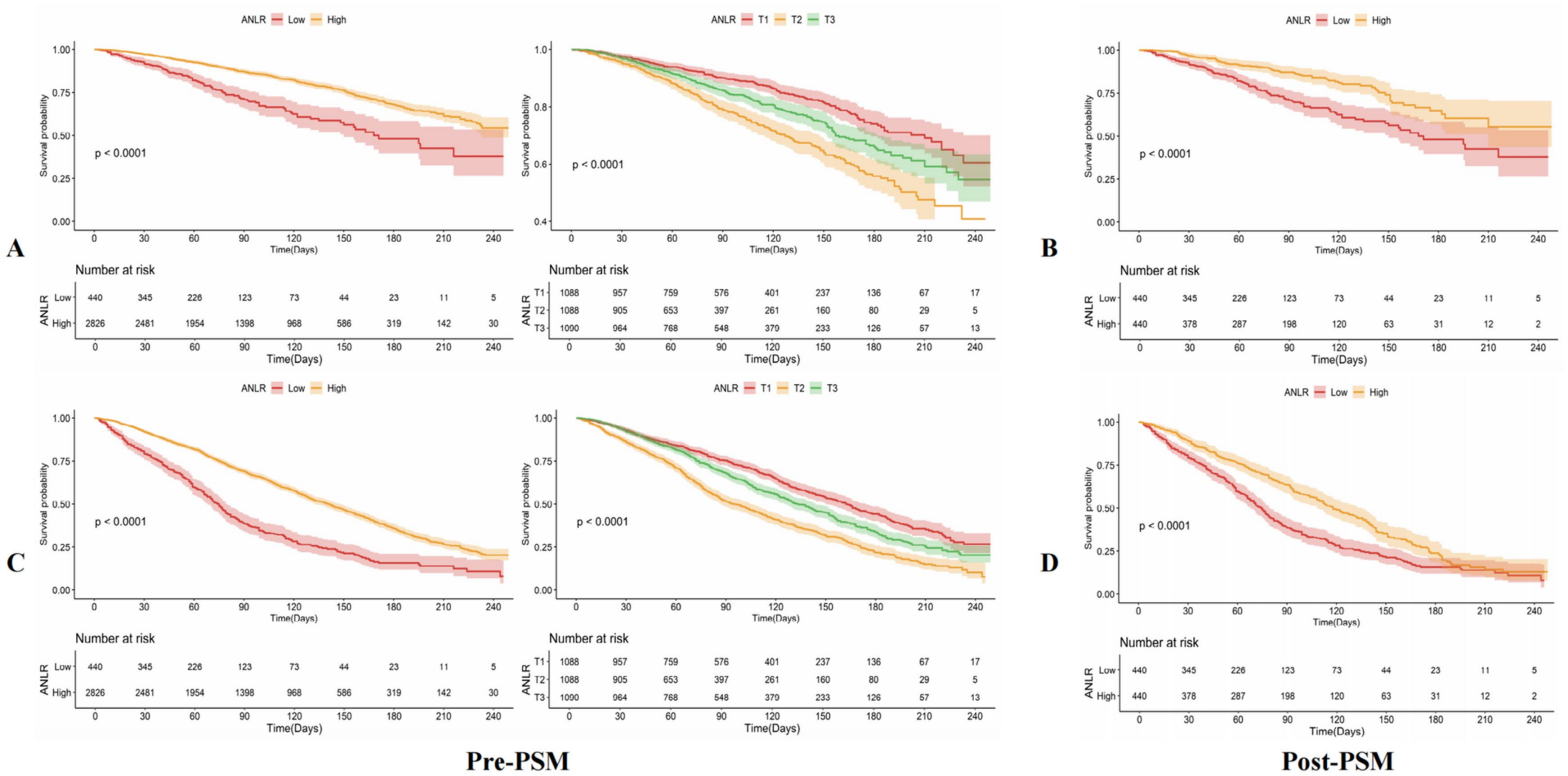
The KM survival curves of **(A,B)** CVM and **(C,D)** ACM pre- and post-PSM.

KM survival curves further validated the differences in mortality outcomes between the low and high ANLR score groups, demonstrating that the lower ANLR group experienced significantly higher CVM and ACM rates compared to those with higher ANLR scores. The survival percentages showed significant disparities: 26.14% *vs.* 17.34% for CVM (p < 0.001) and 68.41% *vs.* 47.17% for ACM (*p* < 0.001). These findings are visually represented in Figures 3A,C.

**Figure 4:**
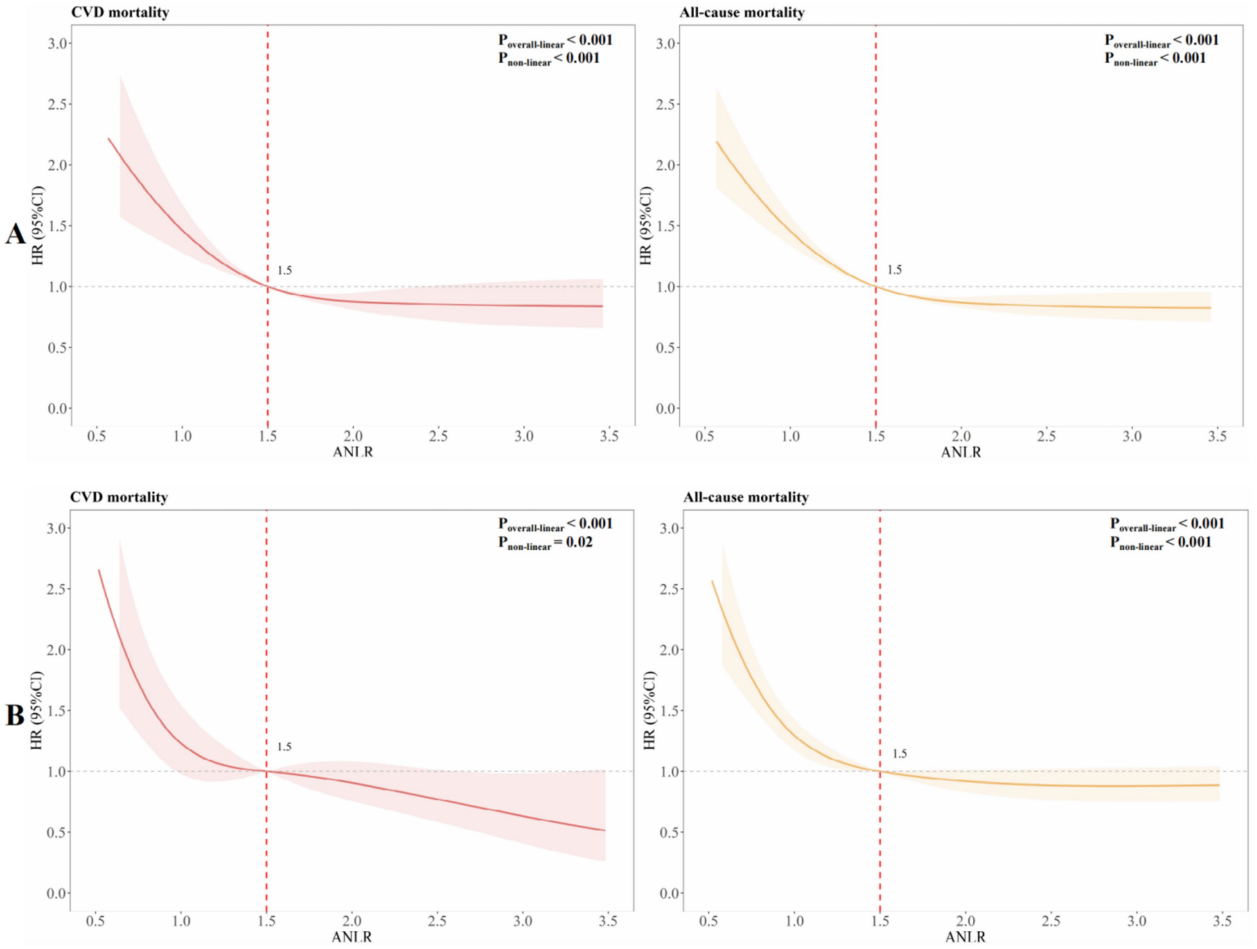
RCSs for CVM and ACM **(A)** pre- and **(B)** post-PSM.

To explore potential non-linear associations, RCS was employed to fit smooth curves using generalized additive models. This analysis aimed to identify the threshold effect of ANLR on CVM and ACM before PSM, highlighting any inflection points. The results revealed an L-shaped non-linear correlation between ANLR and both CVM and ACM pre-PSM (all *p*-values for non-linearity < 0.001, Figure 4A).

### Baseline characteristics of subjects post-PSM

**Figure 5:**
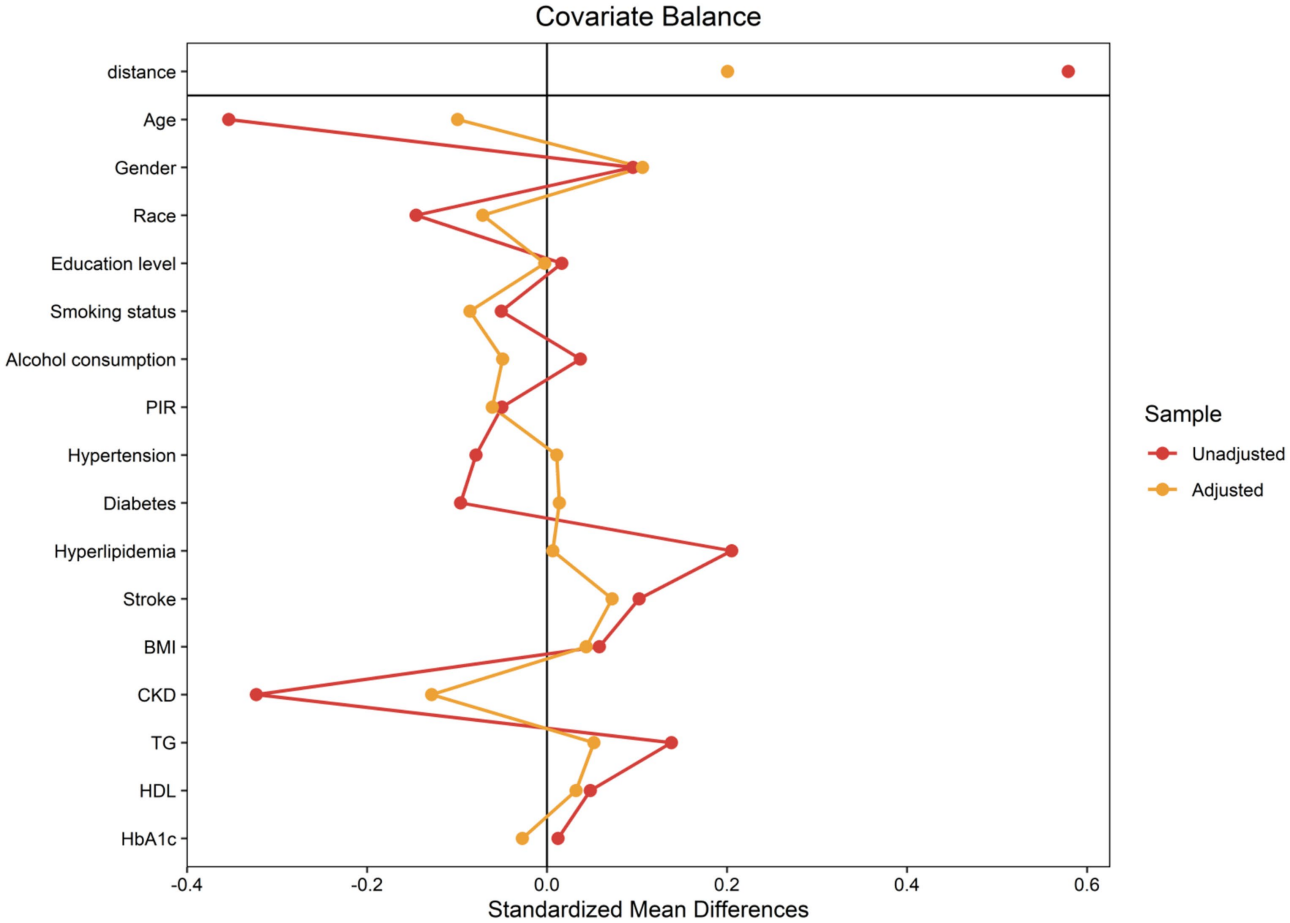
The absolute standardized disparities for the matching variables between both groups.

The application of a 1:1 PSM method aimed at minimizing baseline disparities between the low and high ANLR groups resulted in the successful pairing of 404 patient pairs (Table 3). Figure 5 illustrates the efficacy of the PSM process, as assessed by the measurement of ASD in both pre- and post-PSM. Following the matching procedure, the median age across the two groups became comparable. The proportion of males also became more similar, with 37.05% in the low ANLR group and 42.27% in the high ANLR group. However, ethnic disparities remained to some degree, with a higher percentage of non-Hispanic White in the low ANLR group (74.55% *vs.* 57.05%) and a higher proportion of non-Hispanic Black in the high ANLR group (18.64% *vs.* 9.54%). The groups did not differ significantly in terms of marital status, family PIR, educational attainment, alcohol consumption, smoking status, or BMI following matching. Regarding comorbidities, only MI (*p* = 0.02) and cancer (*p* = 0.003) exhibited significant variation between the groups. Physiological measures showed a notable increase in neutrophil levels in the low ANLR group, while the high ANLR group exhibited higher ALB and lymphocyte counts. No significant differences were observed in TG, HDL, or HbA1c levels. Clinically, individuals in the low ANLR group still had significantly higher rates of CVM (26.14% *vs.* 16.14%, *p* < 0.001) and ACM (68.41% *vs.* 54.55%, *p* < 0.001).

**Table 3:** Baseline characteristics and findings of advanced CKM syndrome individuals post-PSM.

Variables	Overall (*n* = 880)	ANLR		*p-*value
		Low (*n* = 440)	High (*n* = 440)	
ANLR, median (IQR)	1.04 (0.84–1.89)	0.84 (0.69–0.95)	1.89 (1.47–2.53)	< 0.001
Age, years, median (IQR)	76 (69–80)	76.5 (70–80)	76 (67.5–80)	0.13
Sex, male, *n* (%)	349 (39.66)	163 (37.05)	186 (42.27)	0.11
Ethnicity, *n* (%)				<0.001
Mexican American	88 (10.00)	38 (8.64)	50 (11.36)	
Non-Hispanic Black	124 (14.09)	42 (9.54)	82 (18.64)	
Non-Hispanic White	579 (65.80)	328 (74.55)	251 (57.05)	
Other Hispanic	51 (5.80)	18 (4.09)	33 (7.50)	
Other	38 (4.32)	14 (3.18)	24 (5.46)	
Marital status, *n* (%)				0.19
Divorced/Widowed/separated	350 (39.77)	188 (42.73)	162 (36.82)	
Married/Partnered	498 (56.59)	236 (53.64)	262 (59.55)	
Never married	32 (3.64)	16 (3.64)	16 (3.64)	
Education, *n* (%)				0.97
Above HS	360 (40.91)	179 (40.68)	181 (41.14)	
HS or Equivalent	211 (23.98)	107 (24.32)	104 (23.64)	
Below HS	309 (35.11)	154 (35.00)	155 (35.23)	
Family PIR,%, *n* (%)				0.25
<1.30	277 (31.48)	128 (29.09)	149 (33.86)	
1.30–3.49	408 (46.36)	215 (48.86)	193 (43.86)	
≥3.50	195 (22.16)	97 (22.05)	98 (22.27)	
Smoking status, *n* (%)				0.28
Never	339 (38.52)	158 (35.91)	181 (41.14)	
Former	396 (45.00)	207 (47.05)	189 (42.95)	
Now	145 (16.48)	75 (17.05)	70 (15.91)	
Alcohol consumption, *n* (%)				0.40
Never	143 (16.25)	61 (13.86)	82 (18.64)	
Former	307 (34.89)	161 (36.59)	146 (33.18)	
Mild	313 (35.57)	160 (36.36)	153 (34.77)	
Moderate	49 (5.57)	25 (5.68)	24 (5.46)	
Heavy	68 (7.73)	33 (7.50)	35 (7.96)	
Hypertension, *n* (%)	710 (80.68)	354 (80.45)	356 (80.91)	0.86
Diabetes, *n* (%)	427 (48.52)	212 (48.18)	215 (48.86)	0.84
Hyperlipidemia, *n* (%)	689 (78.30)	344 (78.18)	345 (78.41)	0.93
Stroke, *n* (%)	163 (18.52)	75 (17.05)	88 (20.00)	0.26
CHD, *n* (%)	213 (24.2)	115 (26.1)	98 (22.3)	0.18
CHF, *n* (%)	175 (19.9)	106 (24.1)	69 (15.7)	0.002
MI, *n* (%)	209 (23.75)	119 (27.05)	90 (20.45)	0.02
Angina, *n* (%)	130 (14.77)	72 (16.36)	58 (13.18)	0.18
Liver condition, *n* (%)	58 (6.59)	31 (7.04)	27 (6.14)	0.59
CKD, *n* (%)	504 (57.27)	266 (60.45)	238 (54.09)	0.06
CKM, *n* (%)				0.54
*3*	353 (40.11)	172 (39.09)	181 (41.14)	
*4*	527 (59.89)	268 (60.91)	259 (58.86)	
Cancer, *n* (%)	239 (27.16)	139 (31.59)	100 (22.73)	0.003
Data presented as median (IQR)				
BMI, kg/m^2^	28.03 (24.7–32.22)	27.6 (24.21–32.29)	28.3 (25.18–32.18)	0.14
ALB, g/dl	4 (3.8–4.3)	4 (3.8–4.2)	4.1 (3.9–4.4)	< 0.001
Neutrophils, 10^9^/L	4.8 (3.7–6.05)	5.8 (4.8–6.9)	3.9 (3.2–4.7)	< 0.001
Lymphocytes,10^9^/L	1.5 (1.1–1.9)	1.2 (0.9–1.5)	1.8 (1.5–2.3)	< 0.001
TG, mg/dl	117 (81–165.5)	112 (80–165.5)	119.5 (82.5–165.5)	0.34
HDL-C, mg/dl	48 (41–58)	47 (41–58)	49 (42–58)	0.28
HbA1c, %	5.8 (5.4–6.5)	5.8 (5.4–6.5)	5.8 (5.5–6.45)	0.65
CVM, *n* (%)	186 (21.14)	115 (26.14)	71 (16.14)	< 0.001
ACM, *n* (%)	541 (61.48)	301 (68.41)	240 (54.55)	< 0.001

ANLR: albumin/neutrophil to lymphocyte ratio; HS: high school; PIR: poverty-income ratio; CHD: coronary heart disease; CHF: chronic heart failure; MI: myocardial infarction; CKD: chronic kidney disease; CKM: cardiovascular-kidney-metabolic syndrome; BMI: body mass index; ALB: albumin; TG: triglycerides; HDL-C: high-density lipoprotein; hemoglobin A1c: HbA1c; CVM: cardiovascular mortality; ACM: all-cause mortality.

### Associations of the ANLR with mortality outcomes in advanced CKM syndrome participants post-PSM

Table 4 summarizes the outcomes of univariable and multivariable Cox PH regression analyses for CVM and ACM following PSM, using three levels of model adjustment. Regarding CVM, participants with an ANLR score below 1.04 were found to have a significantly higher risk of mortality compared to those with higher ANLR values. In the unadjusted model (Model 1), the HR was 2.07 (95% CI: 1.54–2.78, *p* < 0.001). This elevated risk persisted after adjustment for age, gender, and race/ethnicity in Model 2, with an HR of 1.99 (95% CI: 1.47–2.69, *p* < 0.001), and remained significant in Model 3, which was additionally adjusted for educational level, family PIR, tobacco consumption, alcohol use, hypertension, diabetes, hyperlipidemia, stroke, and BMI, yielding an HR of 2.02 (95% CI: 1.49–2.75, *p* < 0.001). Similarly, for ACM, an ANLR score below 1.04 was related to a significantly higher death risk. In Model 1, the HR was 1.60 (95% CI: 1.35–1.90, *p* < 0.001). This association remained robust subsequent to the adjustment of confounders in Models 2 and 3, with HRs of 1.52 (95% CI: 1.28–1.81, *p* < 0.001) and 1.52 (95% CI: 1.27–1.81, *p* < 0.001), respectively. KM survival curves further established the significantly lower survival rates among participants with an ANLR score below 1.04 compared to those with an ANLR score of 1.04 or higher (Figures 3B,D). RCS revealed an L-shaped non-linear association between ANLR and both CVM and ACM post-PSM (all *p*-values for non-linearity < 0.005, Figure 4B).

**Table 4:** HR (95% CIs) for connection between ANLR and mortality outcomes in advanced CKM syndrome post-PSM.

Outcomes	Models					
	I		II		III	
	HR (95% CI)	*p*-value	HR (95% CI)	*p*-value	HR (95% CI)	*p*-value
CVM						
ANLR (<1.04)	2.07 (1.54–2.78)	<0.001	1.99 (1.47–2.69)	<0.001	2.02 (1.49–2.75)	<0.001
ANLR (≥1.04)	*Ref.*		*Ref.*		*Ref.*	
ACM						
ANLR (< 1.04)	1.60 (1.35–1.90)	<0.001	1.52 (1.28–1.81)	<0.001	1.52 (1.27–1.81)	<0.001
ANLR (≥ 1.04)	*Ref.*		*Ref.*		*Ref.*	

ANLR: albumin/neutrophil to lymphocyte ratio; CVM: cardiovascular mortality; ACM: all-cause mortality.

### Subgroup analysis for the ANLR on CVM and ACM in participants with advanced CKM syndrome

**Figure 6:**
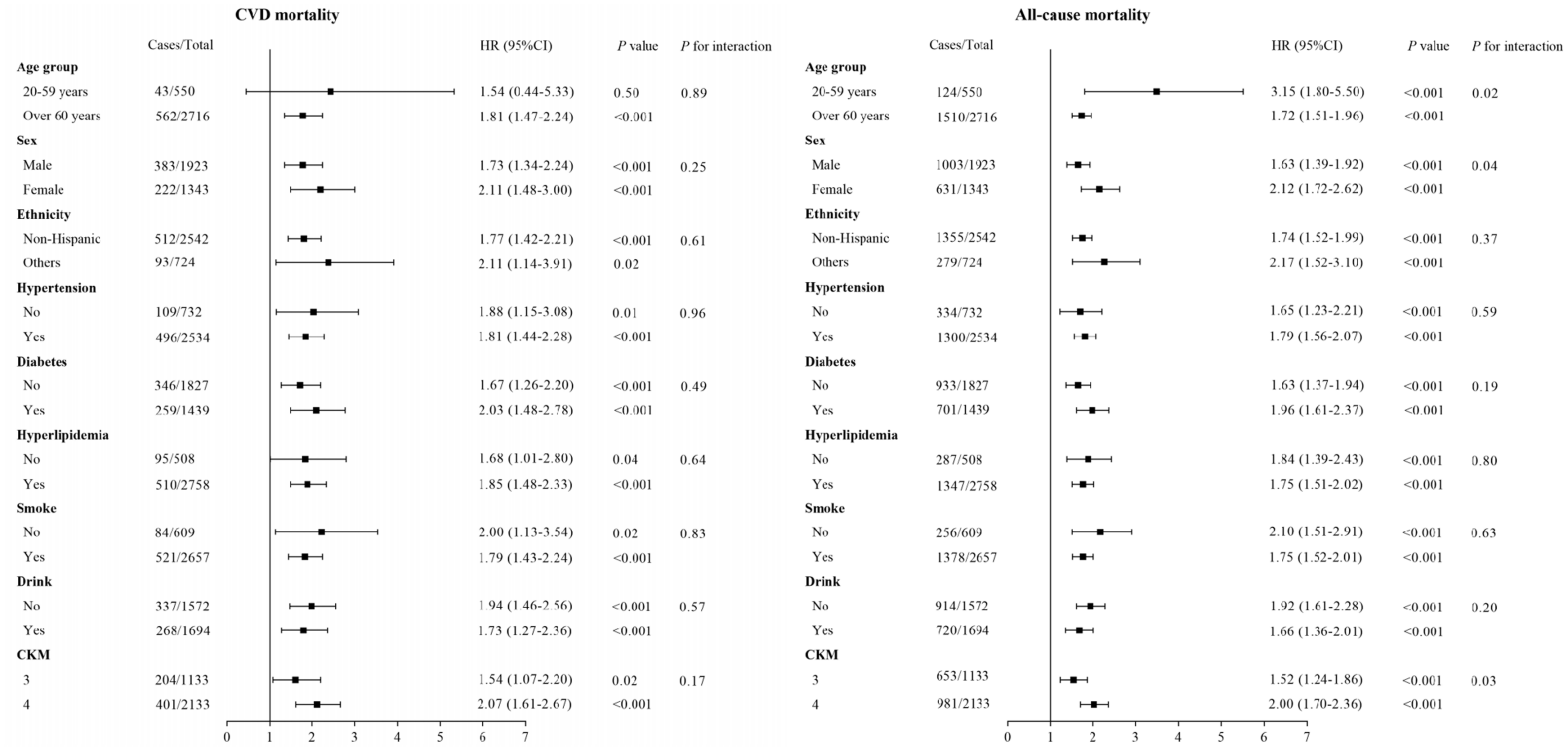
Forest plots of HRs for **(A)** CVM and **(B)** ACM in different subgroups.

Subgroup analyses were carried out to examine the relationship between ANLR scores and CVM and ACM across different categories, including age, sex, race/ethnicity, hypertension, diabetes, hyperlipidemia, smoke, drinking, and CKM stages (Figure 6). The outcomes revealed a significant inverse correlation between ANLR scores and CVM among participants with advanced CKM syndrome in all subgroups, except for those in the 20–59 years age group (*p* = 0.50). Moreover, ANLR and the various subgroup factors did not interact significantly (all *p* > 0.05). Regarding ACM, a similar negative relationship was detected between ANLR scores and mortality in all subgroups of advanced CKM syndrome. However, significant interactions were noted between ANLR and age, gender, and CKM stages (*p* < 0.05).

### Comparison of the predictive performance of ANLR, NLR, and ALB on CVM and ACM in participants with advanced CKM syndrome

An assessment of the predictive value of ANLR, NLR, and ALB for CVM and ACM in individuals with advanced CKM syndrome revealed that the area under the ROC curve (AUC) values for ANLR and NLR for ACM were 0.602 and 0.601, respectively, while the AUC for both ANLR and NLR for CVM was 0.565. These results indicate that although both indices provide some prognostic value, their diagnostic performance is moderate, and they cannot be considered highly effective indicators. ANLR and NLR exhibited comparable predictive capabilities for both ACM and CVM, outperforming ALB (Supplementary Figure S2). Moreover, the predictive performance of ANLR and NLR for ACM was slightly superior to that for CVM (0.602 vs. 0.565).

## Discussion

To date, limited research has focused specifically on advanced CKM syndrome. In this study, we analyzed data from 3,266 individuals with advanced CKM syndrome from NHANES 1999–2018 and found that individuals in the low ANLR group were generally older, had fewer males, and were more often widowed or divorced—features indicative of geriatric vulnerability. These characteristics are associated with frailty, sarcopenia, and reduced nutritional status, as evidenced by their lower serum albumin levels (4.0 vs. 4.2 g/dL). Such demographic factors, including social isolation linked to marital status, may contribute to increased health risks in this population. Additionally, lower levels of ANLR (< 1.04) were significantly associated with increased risks of both CVM and ACM, even after PSM and multivariable adjustment. Notably, we identified an L-shaped non-linear relationship, indicating a steep increase in mortality risk at lower ANLR levels. These associations remained robust across multiple subgroups, suggesting that ANLR may serve as a broadly applicable prognostic marker in high-risk CKM populations.

While traditional CKM biomarkers such as NT-proBNP, troponins, and kidney injury molecule-1 offer mechanistic insights, their clinical use is often limited by high cost, lack of standardization, and restricted availability in primary care. In contrast, ANLR is derived from two low-cost and widely accessible blood markers—serum ALB and white blood cell counts—routinely measured in most clinical settings. This makes ANLR a practical, scalable tool for early identification of high-risk individuals in both general and specialized healthcare environments. Taken together, our findings emphasize the importance of evaluating both nutritional and inflammatory status in predicting outcomes among individuals with advanced CKM syndrome. Moreover, they suggest that systemic inflammation may be a key contributor to the elevated mortality risk in this population. These results provide important insights into the interconnected pathways of cardiovascular, renal, and metabolic dysfunction and highlight the potential of ANLR as a population-level prognostic indicator worthy of further investigation.

Advanced CKM syndrome is a multifaceted multi-organ disorder characterized by failure of the cardiac, renal, and vascular systems. Its pathophysiological mechanisms are intricate, encompassing the interaction of multiple factors such as neurohormones, inflammatory processes, oxidative stress, and metabolic disorders (35). Serum ALB is the major plasma protein produced by the liver and is commonly utilized to evaluate the dietary status of individuals. Clinically, decreased ALB concentrations are often related to malnutrition, inflammatory responses, and organ dysfunction (36, 37). In advanced CKM participants, low ALB levels typically reflect the combined effects of malnutrition and chronic inflammation, both of which have an essential function in the progression of CVDs and renal dysfunction (38, 39). The high prevalence of CKD (60.4%) in this cohort underscores the advanced cardiometabolic and renal burden in individuals with CKM syndrome. CKD is closely associated with systemic inflammation, impaired protein metabolism, and hypoalbuminemia, all of which may lower ANLR and increase mortality risk. These pathophysiological links may partly explain the strong predictive value of low ANLR observed in our study. Investigations illustrated that low ALB concentrations had a positive correlation with ACM and ACM (40, 41), a phenomenon that is particularly prominent in patients with CKD (42). A decrease in ALB is not only related to immune function impairment but may also increase the burden on the cardiovascular system, thereby promoting the occurrence of CVDs (26). A decline in ALB levels reflects malnutrition and systemic inflammation, both of which may accelerate metabolic disturbances and exacerbate the deterioration of cardiac and renal function (43). Malnutrition can lead to insufficient energy supply to the myocardium, increasing cardiac workload, while inflammation participates in the progression of atherosclerosis and CVDs, further raising the risk of mortality (44).

The NLR is a simple and effective marker of the inflammatory response, reflecting the ratio of neutrophils to lymphocytes in the immune system. Recent studies have analyzed similar inflammatory biomarkers in CKD and CVD populations. For example, high NLR has been linked to worsening renal function and elevated cardiovascular risk in CKD cohorts (45, 46). Notably, Rashid et al. reported that higher NLR quartiles were associated with a 1.57-fold increase in mortality among non-dialysis CKD patients (47). Meanwhile, neutrophil percentage to ALB ratio (NPAR), a composite marker combining neutrophil percentage and ALB, has shown superior predictive performance for CVM compared to NLR alone (48). In a large US cohort, elevated NLR was non-linearly associated with approximate doubling of ACM and CVM (49–51). Our findings align well with emerging evidence. The incremental prognostic value of composite biomarkers like NPAR/ANLR over NLR alone has now been documented in CKD populations (46–51). Additionally, the L-shaped survival relationship observed in our cohort mirrors the non-linear risk associations previously described in large-scale NHANES analyses. In advanced CKM syndrome participants, an elevated NLR is often closely associated with a chronic low-grade inflammatory state. An increase in neutrophils indicates the presence of acute or chronic inflammation, while a decrease in lymphocytes suggests that the immune system may be in a state of immunosuppression or functional decline (52). Together, these results reinforce the biological plausibility and potential clinical utility of ANLR in cardiorenal–metabolic risk stratification. Moreover, inflammation has a central function in the onset and progression of CVDs and metabolic diseases. Studies have indicated that elevated NLR is strongly correlated with the occurrence of various CVDs, particularly in patients with diabetes, hypertension, and CKD, and is significantly associated with both CVM and ACM (22, 53–57). As an inflammatory biomarker, an elevated NLR may promote CVDs through multiple mechanisms. For instance, the activation of neutrophils facilitates the formation of atherosclerosis, increasing the vascular inflammatory burden, while a reduction in lymphocytes may impair the anti-inflammatory response, further exacerbating the progression of CVDs (58–62). Additionally, NLR is closely related to factors including insulin resistance and abnormal fat metabolism in MetS, both of which are important risk factors for CVDs (63–65).

Recent mechanistic studies elucidate biological pathways explaining why low ANLR predicts adverse outcomes in advanced CKM syndrome. Miao et al. demonstrated that Sirt6-mediated suppression of intrarenal RAAS/Wnt signaling protects podocytes, mitigating renal inflammation (66). Spatial transcriptomics by Slabowska et al. revealed enhanced infiltration of pro-inflammatory immune cells and dysregulated metabolism in kidney and vascular tissues from CKD/CVD patients (67). Another Miao et al. study showed *Lactobacillus johnsonii* maintains indole-3-aldehyde levels to prevent AHR-driven renal fibrosis (68). Shen et al. found that PPARG/AMPK pathway modulation through herbal compounds restores metabolic balance and reduces CKD–MBD pathology (69). Finally, *L. johnsonii* supplementation improved renal and gut barrier integrity via IAld–AHR suppression in CKD models (70). Together, these findings underscore that low ANLR reflects a complex state of inflammation, malnutrition, dysregulated RAAS, microbial dysbiosis, and transcriptomic–metabolic dysfunction, providing mechanistic justification for its prognostic relevance in CKM syndrome.

Both serum ALB and NLR independently reflect the clinical condition of patients as biomarkers, but their combined use may provide a more accurate prognosis. Several studies have explored composite biomarkers combining ALB with leukocyte parameters, yielding promising prognostic value across clinical conditions. For instance, the ALB- × -NLR score in head and neck cancer patients demonstrated superior ability to predict chemoradiotherapy tolerance and adverse events compared to either marker alone (71). Similarly, in nasopharyngeal carcinoma, the lymphocyte–ALB–neutrophil ratio served as an independent predictor of overall and progression-free survival and was incorporated into validated prognostic nomograms (C-index = 0.70) (72). In non-cancer cohorts, the NPAR exhibited a non-linear association with ACM and CVM in metabolic dysfunction-associated steatotic liver disease patients, outperforming traditional NLR in predictive performance (73). Moreover, among septic shock patients, the neutrophil-to-lymphocyte-to-ALB ratio achieved an AUC of 0.70 in predicting in-hospital mortality, surpassing conventional severity scores (74). In advanced CKM syndrome participants, low ALB and high NLR often coexist, representing severe malnutrition and significant inflammatory responses. Study have pointed out that the joint assessment of ALB and NLR can predict the risk of diabetic foot ulcers (29). The combination of low ALB and high NLR may signal a persistent inflammatory state and poor nutritional status in patients (29). These factors interact to accelerate disease progression, ultimately increasing mortality. Therefore, serum ALB and NLR not only provide important predictive value for the prognosis of advanced CKM syndrome participants when assessed individually, but their combined use offers a more precise clinical evaluation, helping to detect high-risk individuals and guide personalized treatment approaches.

The study highlights significant findings, including the utilization of a large, nationwide representative sample of US individuals that was followed longitudinally and accounted for most possible confounders. We emphasize the potential of the ANLR as a predictive indicator for death risk in advanced CKM syndrome participants, as this index can be easily derived from a standard complete blood count and routine biochemical tests without additional costs. It can help doctors identify high mortality risk advanced CKM syndrome participants and implement targeted therapies to reduce risks. Moreover, we used a 1:1 PSM technique to improve the validity of our results and conducted subgroup analyses to validate the strength of the observed correlations.

Several limitations must be acknowledged. First, although NHANES provides longitudinal mortality follow-up, its cross-sectional design limits our ability to capture the timing of biomarker measurements and disease progression, restricting our capacity to assess time-varying exposures or infer causality. Second, the single baseline assessment of ANLR prevents evaluation of its changes over time. The ANLR cutoff identified in this study is exploratory and data-driven; while internal validation supports its stability, external validation in independent cohorts is needed before clinical adoption. Additionally, the AUC values for ANLR and NLR in predicting ACM and CVM were between 0.5 and 0.6, indicating moderate diagnostic efficiency, necessitating refinement of these indices in future research. Third, while we accounted for most confounding variables, residual confounding from unmeasured factors like food habits, genetic predisposition, and psychological stress remains a concern. Moreover, we lacked data on acute inflammation and medication use, which could have influenced leukocyte counts. The interdependence of ANLR components limits their simultaneous use as mortality predictors. Fourth, our adaptation of CKM staging was based on NHANES data, which lacks diagnostic tools such as CAC scores and NT-proBNP. Subclinical cardiovascular conditions were approximated through ASCVD risk estimates. Fifth, clinical CVDs were self-reported, which may underestimate conditions like PAD and AF. We also could not stratify CKM stages 4a and 4b due to sample size limitations. Lastly, further research is needed to validate these findings, explore underlying mechanisms, and determine if increasing ANLR could lower mortality in advanced CKM syndrome patients.

Future studies should aim to validate these findings in prospective CKM cohorts with longitudinal biomarker data and clinical endpoints. Additionally, mechanistic investigations integrating gut microbiota profiles, transcriptomics, and immune-inflammatory signatures may help clarify the biological pathways linking ANLR to adverse outcomes. Finally, evaluating whether ANLR can enhance existing CKM risk prediction models or guide targeted interventions will be important for its clinical translation.

## Conclusion

Overall, lower ANLR values, which indicate heightened systemic inflammation and malnutrition, were shown to be independently connected with a greater death risk from ACM and CVM in US advanced CKM syndrome participants. These readily accessible and affordable blood indices may be valuable prognostic indicators for identifying high mortality risk advanced CKM syndrome persons. Future research should focus on incorporating additional biomarkers, validating the indices in larger and more diverse cohorts, and employing advanced analytical methods to refine the diagnostic efficiency of ANLR and NLR for better clinical utility.

## Data Availability

The raw data supporting the conclusions of this article will be made available by the authors, without undue reservation.

## References

[1] Ndumele CE Neeland IJ Tuttle KR Chow SL Mathew RO Khan SS. A synopsis of the evidence for the science and clinical Management of Cardiovascular-Kidney-Metabolic (CKM) syndrome: a scientific statement from the American Heart Association. Circulation. (2023) 148:1636–64. doi: 10.1161/CIR.0000000000001186, PMID: 37807920

[2] Marassi M Fadini GP. The cardio-renal-metabolic connection: a review of the evidence. Cardiovasc Diabetol. (2023) 22:195. doi: 10.1186/s12933-023-01937-x, PMID: 37525273 PMC10391899

[3] Sebastian SA Padda I Johal G. Cardiovascular-kidney-metabolic (CKM) syndrome: a state-of-the-art review. Curr Probl Cardiol. (2024) 49:102344. doi: 10.1016/j.cpcardiol.2023.102344, PMID: 38103820

[4] Zhu R Wang R He J Wang L Chen H Niu X. Prevalence of cardiovascular-kidney-metabolic syndrome stages by social determinants of health. JAMA Netw Open. (2024) 7:e2445309. doi: 10.1001/jamanetworkopen.2024.45309, PMID: 39556396 PMC11574692

[5] Zhao BR Hu XR Wang WD Zhou Y. Cardiorenal syndrome: clinical diagnosis, molecular mechanisms and therapeutic strategies. Acta Pharmacol Sin. (2025) 46:1539–55. doi: 10.1038/s41401-025-01476-z, PMID: 39910210 PMC12098865

[6] Zhang J Zhu P Li S Gao Y Xing Y. From heart failure and kidney dysfunction to cardiorenal syndrome: TMAO may be a bridge. Front Pharmacol. (2023) 14:1291922. doi: 10.3389/fphar.2023.1291922, PMID: 38074146 PMC10703173

[7] Zhu T Du Y Xuan M Guo C Rao X. Clinical characteristics and Chinese medicine therapy of chronic kidney disease combined with cardiovascular disease. Integr Med Nephrol Androl. (2023) 10:e00023. doi: 10.1097/IMNA-D-22-00023

[8] Taheri S. Heterogeneity in cardiorenal protection by sodium glucose cotransporter 2 inhibitors in heart failure across the ejection fraction strata: systematic review and meta-analysis. World J Nephrol. (2023) 12:182–200. doi: 10.5527/wjn.v12.i5.182, PMID: 38230296 PMC10789083

[9] Wang YN Miao H Yu XY Guo Y Su W Liu F. Oxidative stress and inflammation are mediated via aryl hydrocarbon receptor signalling in idiopathic membranous nephropathy. Free Radic Biol Med. (2023) 207:89–106. doi: 10.1016/j.freeradbiomed.2023.07.014, PMID: 37451370

[10] Chen Y Guo JB Peng W Xiang XY Wang YF Luo Y. Treatment of COVID-19-induced systematic inflammatory response and multiple organ failure using Xuebijing. Integr Med Nephrol Androl. (2023) 10:e00018. doi: 10.1097/IMNA-D-22-00018

[11] Li H Li L. Inverse associations of the lifestyle critical 9 with cardiorenal syndrome: the mediating role of the dietary inflammatory index. Front Nutr. (2025) 12:1519612. doi: 10.3389/fnut.2025.1519612, PMID: 40151350 PMC11948285

[12] Libby P Ridker PM Hansson GK Leducq Transatlantic Network on Atherothrombosis. Inflammation in atherosclerosis: from pathophysiology to practice. J Am Coll Cardiol. (2009) 54:2129–38. doi: 10.1016/j.jacc.2009.09.009, PMID: 19942084 PMC2834169

[13] Attiq A Afzal S Ahmad W Kandeel M. Hegemony of inflammation in atherosclerosis and coronary artery disease. Eur J Pharmacol. (2024) 966:176338. doi: 10.1016/j.ejphar.2024.176338, PMID: 38242225

[14] Tonelli M Sacks F Pfeffer M Jhangri GS Curhan G Cholesterol and Recurrent Events (CARE) Trial Investigators. Biomarkers of inflammation and progression of chronic kidney disease. Kidney Int. (2005) 68:237–45. doi: 10.1111/j.1523-1755.2005.00398.x, PMID: 15954913

[15] Hotamisligil GS. Inflammation and metabolic disorders. Nature. (2006) 444:860–7. doi: 10.1038/nature05485, PMID: 17167474

[16] Dere Ö Dere Y. The impact of hematologic parameters on histopathologic features of colorectal cancer. Int J Gen Med. (2024) 17:2029–36. doi: 10.2147/IJGM.S463588, PMID: 38741678 PMC11090114

[17] Guo X Tang J He H Jian L Qiang O Xie Y. Body composition and inflammation variables as the potential prognostic factors in epithelial ovarian cancer treated with Olaparib. Front Oncol. (2024) 14:1359635. doi: 10.3389/fonc.2024.1359635, PMID: 38725625 PMC11079183

[18] Li J Wang X Jia W Wang K Wang W Diao W. Association of the systemic immuno-inflammation index, neutrophil-to-lymphocyte ratio, and platelet-tolymphocyte ratio with diabetic microvascular complications. Front Endocrinol. (2024) 15:1367376. doi: 10.3389/fendo.2024.1367376, PMID: 38660516 PMC11039910

[19] Du P Gao X Sun Q Gong M Pan Y Guo Q. Association between uric acid and cardiac outcomes mediated by neutrophil-to-lymphocyte ratio in patients with left ventricular diastolic dysfunction and pulmonary hypertension. Sci Rep. (2024) 14:2751. doi: 10.1038/s41598-024-53077-1, PMID: 38302600 PMC10834525

[20] Kim S Eliot M Koestler DC Wu WC Kelsey KT. Association of Neutrophil-to-Lymphocyte Ratio with Mortality and Cardiovascular Disease in the Jackson heart study and modification by the Duffy antigen variant. JAMA Cardiol. (2018) 3:455–62. doi: 10.1001/jamacardio.2018.1042, PMID: 29801037 PMC6128503

[21] Wang X Zhang G Jiang X Zhu H Lu Z Xu L. Neutrophil to lymphocyte ratio in relation to risk of all-cause mortality and cardiovascular events among patients undergoing angiography or cardiac revascularization: a meta-analysis of observational studies. Atherosclerosis. (2014) 234:206–13. doi: 10.1016/j.atherosclerosis.2014.03.003, PMID: 24681815

[22] Dong G Gan M Xu S Xie Y Zhou M Wu L. The neutrophil-lymphocyte ratio as a risk factor for all-cause and cardiovascular mortality among individuals with diabetes: evidence from the NHANES 2003-2016. Cardiovasc Diabetol. (2023) 22:267. doi: 10.1186/s12933-023-01998-y, PMID: 37775767 PMC10541705

[23] Viers BR Boorjian SA Frank I Tarrell RF Thapa P Karnes RJ. Pretreatment neutrophil-to-lymphocyte ratio is associated with advanced pathologic tumor stage and increased cancer-specific mortality among patients with urothelial carcinoma of the bladder undergoing radical cystectomy. Eur Urol. (2014) 66:1157–64. doi: 10.1016/j.eururo.2014.02.042, PMID: 24630414

[24] Artigas A Wernerman J Arroyo V Vincent J-L Levy M. Role of albumin in diseases associated with severe systemic inflammation: pathophysiologic and clinical evidence in sepsis and in decompensated cirrhosis. J Crit Care. (2016) 33:62–70. doi: 10.1016/j.jcrc.2015.12.019, PMID: 26831575

[25] Thuemmler RJ Pana TA Carter B Mahmood R Bettencourt-Silva JH Metcalf AK. Serum albumin and post-stroke outcomes: analysis of UK regional registry data, systematic review, and Meta-analysis. Nutrients. (2024) 16:1486. doi: 10.3390/nu16101486, PMID: 38794724 PMC11124370

[26] Manolis AA Manolis TA Melita H Mikhailidis DP Manolis AS. Low serum albumin: a neglected predictor in patients with cardiovascular disease. Eur J Intern Med. (2022) 102:24–39. doi: 10.1016/j.ejim.2022.05.004, PMID: 35537999

[27] Yang ZC Zhang L Xi YB Jiang GH Lin H Pan H. Association between albumin changes and prognosis in older patients with acute myocardial infarction. Front Med. (2025) 11:1508868. doi: 10.3389/fmed.2024.1508868, PMID: 39902029 PMC11788366

[28] Yang K Yang N Sun W Dai L Jin J Wu J. The association between albumin and mortality in patients with acute kidney injury: a retrospective observational study. BMC Nephrol. (2023) 24:332. doi: 10.1186/s12882-023-03323-x, PMID: 37946135 PMC10636863

[29] Lin Z Zhuang W Wang L Lan W. Association between nutritional inflammation index and diabetic foot ulcers: a population-based study. Front Nutr. (2025) 12:1532131. doi: 10.3389/fnut.2025.1532131, PMID: 39927281 PMC11802432

[30] NHANES questionnaires, datasets, and related documentation. Available online at: https://wwwn.cdc.gov/nchs/nhanes/Default.aspx (Accessed May 25 2024)

[31] Hothorn T Lausen B. Maximally selected rank statistics in R. R News. (2002) 2:3–5.

[32] Aggarwal R Ostrominski JW Vaduganathan M. Prevalence of cardiovascular-kidney metabolic syndrome stages in US adults, 2011–2020. JAMA. (2024) 331:1858–60. doi: 10.1001/jama.2024.6892, PMID: 38717747 PMC11079779

[33] Kidney disease: improving global outcomes CKDWG. KDIGO 2024 clinical practice guideline for the evaluation and management of chronic kidney disease. Kidney Int. (2024) 105:S117–34. doi: 10.1016/j.kint.2023.10.018 38490803

[34] Johnson CL Paulose-Ram R Ogden CL Carroll MD Kruszon-Moran D Dohrmann SM National health and nutrition examination survey: Analytic guidelines, 1999–2010. Vital health stat 2. (2013) 1–24. 25090154

[35] Savira F Magaye R Liew D Reid C Kelly DJ Kompa AR. Cardiorenal syndrome: multi-organ dysfunction involving the heart, kidney and vasculature. Br J Pharmacol. (2020) 177:2906–22. doi: 10.1111/bph.15065, PMID: 32250449 PMC7280015

[36] Eckart A Struja T Kutz A Baumgartner A Baumgartner T Zurfluh S. Relationship of nutritional status, inflammation, and serum albumin levels during acute illness: a prospective study. Am J Med. (2020) 133:713–722.e7. doi: 10.1016/j.amjmed.2019.10.031, PMID: 31751531

[37] Akirov A Masri-Iraqi H Atamna A Shimon I. Low albumin levels are associated with mortality risk in hospitalized patients. Am J Med. (2017) 130:1465.e11–9. doi: 10.1016/j.amjmed.2017.07.020, PMID: 28803138

[38] Jagadeswaran D Indhumathi E Hemamalini AJ Sivakumar V Soundararajan P Jayakumar M. Inflammation and nutritional status assessment by malnutrition inflammation score and its outcome in pre-dialysis chronic kidney disease patients. Clin Nutr. (2019) 38:341–7. doi: 10.1016/j.clnu.2018.01.001, PMID: 29398341

[39] Arques S. Human serum albumin in cardiovascular diseases. Eur J Intern Med. (2018) 52:8–12. doi: 10.1016/j.ejim.2018.04.014, PMID: 29680174

[40] Yang C Lu J Shen F Xie H Cui H Xu R. Serum albumin level is associated with mortality and hospital stays: a real-world data analysis. Clin Nutr ESPEN. (2024) 64:215–20. doi: 10.1016/j.clnesp.2024.10.002, PMID: 39396704

[41] Pan H Lin S. Association of hemoglobin, albumin, lymphocyte, and platelet score with risk of cerebrovascular, cardiovascular, and all-cause mortality in the general population: results from the NHANES 1999-2018. Front Endocrinol. (2023) 14:1173399. doi: 10.3389/fendo.2023.1173399, PMID: 37424853 PMC10328756

[42] Huang F Fan J Wan X Liu H Shi Y Shu H. The association between blood albumin level and cardiovascular complications and mortality risk in ICU patients with CKD. BMC Cardiovasc Disord. (2022) 22:322. doi: 10.1186/s12872-022-02763-x, PMID: 35850629 PMC9295487

[43] Martin-Taboada M Vila-Bedmar R Medina-Gómez G. From obesity to chronic kidney disease: how can adipose tissue affect renal function? Nephron. (2021) 145:609–13. doi: 10.1159/000515418, PMID: 33882488

[44] Noels H Lehrke M Vanholder R Jankowski J. Lipoproteins and fatty acids in chronic kidney disease: molecular and metabolic alterations. Nat Rev Nephrol. (2021) 17:528–42. doi: 10.1038/s41581-021-00423-5, PMID: 33972752

[45] Ko HL Jung J Lee J Lim JH Im DW Kim YC. Dynamic nature and prognostic value of the neutrophil-to-lymphocyte ratio in critically ill patients with acute kidney injury on continuous renal replacement therapy: a multicenter cohort study. Front Med. (2023) 10:1162381. doi: 10.3389/fmed.2023.1162381, PMID: 37056733 PMC10086237

[46] Gao W Wang X Zou Y Wang S Dou J Qian S. Progress in the application of novel inflammatory indicators in chronic kidney disease. Front Med. (2025) 12:1500166. doi: 10.3389/fmed.2025.1500166, PMID: 39950124 PMC11821595

[47] Rashid I Tiwari P D’Cruz S Jaswal S. Prognostic importance of neutrophil-lymphocyte ratio in non-dialysis chronic kidney disease patients—a hospital-based prospective cohort. Explor Med. (2023) 4:299–313. doi: 10.37349/emed.2023.00141

[48] Lau LFS Ng JKC Fung WWS Chan GCK Cheng PM Chow KM. Relationship between serial serum neutrophil-lymphocyte ratio, cardiovascular mortality, and all-cause mortality in Chinese peritoneal dialysis patients. Kidney Blood Press Res. (2023) 48:414–23. doi: 10.1159/000530554, PMID: 37166323 PMC10308529

[49] Chu Q Wu B Zhang Z. Association of neutrophil to lymphocyte ratio with all-cause and cardiovascular mortality among individuals with kidney stone disease: result from NHANES, 2007-2018. Front Endocrinol. (2025) 16:1537403. doi: 10.3389/fendo.2025.1537403, PMID: 40162317 PMC11949785

[50] He JM Yang Y. Association between neutrophil-lymphocyte ratio and all-cause and cardiovascular mortality in patients with diabetes or prediabetes with comorbid obstructive sleep apnea symptoms: evidence from NHANES 2005-2008 and 2015-2018. Front Endocrinol. (2025) 16:1512621. doi: 10.3389/fendo.2025.1512621, PMID: 40331136 PMC12052538

[51] Li X Wang L Liu M Zhou H Xu H. Association between neutrophil-to-lymphocyte ratio and diabetic kidney disease in type 2 diabetes mellitus patients: a cross-sectional study. Front Endocrinol. (2024) 14:1285509. doi: 10.3389/fendo.2023.1285509, PMID: 38239986 PMC10795842

[52] Chen Y Guan M Wang R Wang X. Relationship between advanced lung cancer inflammation index and long-term all-cause, cardiovascular, and cancer mortality among type 2 diabetes mellitus patients: NHANES, 1999-2018. Front Endocrinol. (2023) 14:1298345. doi: 10.3389/fendo.2023.1298345, PMID: 38111710 PMC10726345

[53] Zhang X Wei R Wang X Zhang W Li M Ni T. The neutrophil-to-lymphocyte ratio is associated with all-cause and cardiovascular mortality among individuals with hypertension. Cardiovasc Diabetol. (2024) 23:117. doi: 10.1186/s12933-024-02191-5, PMID: 38566082 PMC10985955

[54] Afari ME Bhat T. Neutrophil to lymphocyte ratio (NLR) and cardiovascular diseases: an update. Expert Rev Cardiovasc Ther. (2016) 14:573–7. doi: 10.1586/14779072.2016.1154788, PMID: 26878164

[55] Li X Liu M Wang G. The neutrophil-lymphocyte ratio is associated with all-cause and cardiovascular mortality in cardiovascular patients. Sci Rep. (2024) 14:26692. doi: 10.1038/s41598-024-76836-6, PMID: 39496711 PMC11535400

[56] Liu W Weng S Cao C Yi Y Wu Y Peng D. Association between monocyte-lymphocyte ratio and all-cause and cardiovascular mortality in patients with chronic kidney diseases: a data analysis from national health and nutrition examination survey (NHANES) 2003-2010. Ren Fail. (2024) 46:2352126. doi: 10.1080/0886022X.2024.2352126, PMID: 38832474 PMC11151800

[57] Song S Chen L Yu R Zhu J. Neutrophil-to-lymphocyte ratio as a predictor of all-cause and cardiovascular mortality in coronary heart disease and hypertensive patients: a retrospective cohort study. Front Endocrinol. (2024) 15:1442165. doi: 10.3389/fendo.2024.1442165, PMID: 39234507 PMC11371692

[58] Silvestre-Roig C Braster Q Ortega-Gomez A Soehnlein O. Neutrophils as regulators of cardiovascular inflammation. Nat Rev Cardiol. (2020) 17:327–40. doi: 10.1038/s41569-019-0326-7, PMID: 31996800

[59] Zhao Z Pan Z Zhang S Ma G Zhang W Song J. Neutrophil extracellular traps: a novel target for the treatment of stroke. Pharmacol Ther. (2023) 241:108328. doi: 10.1016/j.pharmthera.2022.108328, PMID: 36481433

[60] Drechsler M Megens RT van Zandvoort M Weber C Soehnlein O. Hyperlipidemia-triggered neutrophilia promotes early atherosclerosis. Circulation. (2010) 122:1837–45. doi: 10.1161/CIRCULATIONAHA.110.961714, PMID: 20956207

[61] Jones DP True HD Patel J. Leukocyte trafficking in cardiovascular disease: insights from experimental models. Mediat Inflamm. (2017) 2017:9746169–9. doi: 10.1155/2017/9746169, PMID: 28465628 PMC5390637

[62] Poller WC Nahrendorf M Swirski FK. Hematopoiesis and cardiovascular disease. Circ Res. (2020) 126:1061–85. doi: 10.1161/CIRCRESAHA.120.315895, PMID: 32271679 PMC7153537

[63] Jornayvaz FR Samuel VT Shulman GI. The role of muscle insulin resistance in the pathogenesis of atherogenic dyslipidemia and nonalcoholic fatty liver disease associated with the metabolic syndrome. Annu Rev Nutr. (2010) 30:273–90. doi: 10.1146/annurev.nutr.012809.104726, PMID: 20645852 PMC3730129

[64] Islam MS Wei P Suzauddula M Nime I Feroz F Acharjee M. The interplay of factors in metabolic syndrome: understanding its roots and complexity. Mol Med. (2024) 30:279. doi: 10.1186/s10020-024-01019-y, PMID: 39731011 PMC11673706

[65] Reddy P Lent-Schochet D Ramakrishnan N McLaughlin M Jialal I. Metabolic syndrome is an inflammatory disorder: a conspiracy between adipose tissue and phagocytes. Clin Chim Acta. (2019) 496:35–44. doi: 10.1016/j.cca.2019.06.019, PMID: 31229566

[66] Miao H Wang YN Su W Zou L Zhuang SG Yu XY. Sirtuin 6 protects against podocyte injury by blocking the renin-angiotensin system by inhibiting the Wnt1/β-catenin pathway. Acta Pharmacol Sin. (2024) 45:137–49. doi: 10.1038/s41401-023-01148-w, PMID: 37640899 PMC10770168

[67] Slabowska AO Pyke C Hvid H Jessen LE Baumgart S Das V. A systematic evaluation of state-of-the-art deconvolution methods in spatial transcriptomics: insights from cardiovascular disease and chronic kidney disease. Front Bioinform. (2024) 4:1352594. doi: 10.3389/fbinf.2024.1352594, PMID: 38601476 PMC11004278

[68] Miao H Wang YN Yu XY Zou L Guo Y Su W. Lactobacillus species ameliorate membranous nephropathy through inhibiting the aryl hydrocarbon receptor pathway via tryptophan-produced indole metabolites. Br J Pharmacol. (2024) 181:162–79. doi: 10.1111/bph.16219, PMID: 37594378

[69] Shen J Liu Y Wang Q Chen H Hu Y Guo X. Integrated network pharmacology, transcriptomics, and metabolomics analysis to reveal the mechanism of salt Eucommiae cortex in the treatment of chronic kidney disease mineral bone disorders via the PPARG/AMPK signaling pathway. J Ethnopharmacol. (2023) 314:116590. doi: 10.1016/j.jep.2023.116590, PMID: 37207881

[70] Miao H Liu F Wang YN Yu XY Zhuang S Guo Y. Targeting *Lactobacillus johnsonii* to reverse chronic kidney disease. Signal Transduct Target Ther. (2024) 9:195. doi: 10.1038/s41392-024-01913-1, PMID: 39098923 PMC11298530

[71] Hsu CC Chou WC Hung YS Lin SY Hung CY Yeh KY. Predictive value of albumin and neutrophil-to-lymphocyte ratio score for treatment completeness and safety profiles in patients with head and neck Cancer receiving definitive concurrent Chemoradiotherapy. In Vivo. (2022) 36:2875–83. doi: 10.21873/invivo.13028, PMID: 36309354 PMC9677765

[72] Zhang S Chen Z Ling J Feng Y Xie Y Liu X. Nomograms based on the lymphocyte-albumin-neutrophil ratio (LANR) for predicting the prognosis of nasopharyngeal carcinoma patients after definitive radiotherapy. Sci Rep. (2024) 14:5388. doi: 10.1038/s41598-024-56043-z, PMID: 38443675 PMC10915143

[73] Dong K Zheng Y Wang Y Guo Q. Predictive role of neutrophil percentage-to-albumin ratio, neutrophil-to-lymphocyte ratio, and systemic immune-inflammation index for mortality in patients with MASLD. Sci Rep. (2024) 14:30403. doi: 10.1038/s41598-024-80801-8, PMID: 39638820 PMC11621551

[74] Thang NVV Luyen LT Vi NTT Hai PD. Neutrophil-to-lymphocyte-to-albumin ratio as a prognostic marker for mortality in sepsis and septic shock in Vietnam. Acute Crit Care. (2025) 40:244–51. doi: 10.4266/acc.003576, PMID: 40494596 PMC12151731

