# Supplementary material for: Altered albumin/neutrophil to lymphocyte ratio are associated with all-cause and cardiovascular mortality for advanced cardiovascular-kidney-metabolic syndrome
Source: Front Nutr. 2025 Jul 16;12:1595119. doi: 10.3389/fnut.2025.1595119 (PMC12307165; doi:10.3389/fnut.2025.1595119)

**Supplementary Table S1.** CKM-NHANES Variable Mapping Table

| **CKM Stage** | **Criteria** | **NHANES Variables** |
| --- | --- | --- |
| **Stage 0** | **Normal BMI** (<25 kg/m²) | BMI (Body Mass Index) |
|  | **Normal Waist Circumference** (Women: <88 cm, Men: <102 cm) | Waist Circumference (WC) |
|  | **Normoglycemia** (FBG <100 mg/dL, HbA1c <5.7%) | Fasting Blood Glucose (FBG), HbA1c (Hemoglobin A1c) |
|  | **Normotension** (Systolic BP <130 mmHg, Diastolic BP <80 mmHg) | Blood Pressure (BP) |
|  | **Normal Lipid Profile** (Triglycerides <135 mg/dL) | Triglycerides (TG) |
|  | **No CKD or CVD** | eGFR (Estimated Glomerular Filtration Rate), CKD (Chronic Kidney Disease) Indicators, CVD Indicators |
| **Stage 1** | **Elevated BMI** (≥25 kg/m²) | BMI |
|  | **Increased Waist Circumference** (Women: ≥88 cm, Men: ≥102 cm) | Waist Circumference (WC) |
|  | **Prediabetes** (HbA1c 5.7%-6.4% or FBG 100-125 mg/dL) | HbA1c, FBG |
| **Stage 2** | **Metabolic Risk Factors** | elevated triglycerides (≥135 mg/dL), hypertension, diabetes, or metabolic syndrome (≥3 of the following: elevated waist circumference, low HDL [<40 mg/dL for men, <50 mg/dL for women], elevated triglycerides [≥150 mg/dL], elevated BP [systolic ≥130 mmHg, diastolic ≥80 mmHg], or prediabetes). |
|  | **Moderate-to-High-Risk CKD** (per KDIGO guidelines)  eGFR: 30-59 ml/min/1.73m²; UACR: 30-300 mg/g | eGFR, UACR |
| **Stage 3** | **Very-High-Risk CKD** (Stage G4 or G5 CKD, eGFR < 30 mL/min/1.73 m²; UACR: > 300 mg/g) or High 10-year CVD Risk** (≥ 20%) | eGFR, UACR  CVD Risk Calculations (AHA PREVENT) |
| **Stage 4** | **Self-Reported Established CVD** (Coronary Heart Disease, Angina, MI, Heart Failure, Stroke) | Self-reported CVD (CHD, Angina, MI, Heart Failure, Stroke) |

1. Ndumele CE, Neeland IJ, Tuttle KR, Chow SL, Mathew RO, Khan SS, et al. A Synopsis of the Evidence for the Science and Clinical Management of Cardiovascular-Kidney-Metabolic (CKM) Syndrome: A Scientific Statement From the American Heart Association. Circulation. 2023;148(20):1636-64.

2. KDIGO 2021 Clinical Practice Guideline for the Management of Glomerular Diseases. Kidney Int. 2021;100(4s):S1-s276.

**Figure S1.** Bootstrap distribution of ANLR cut-point


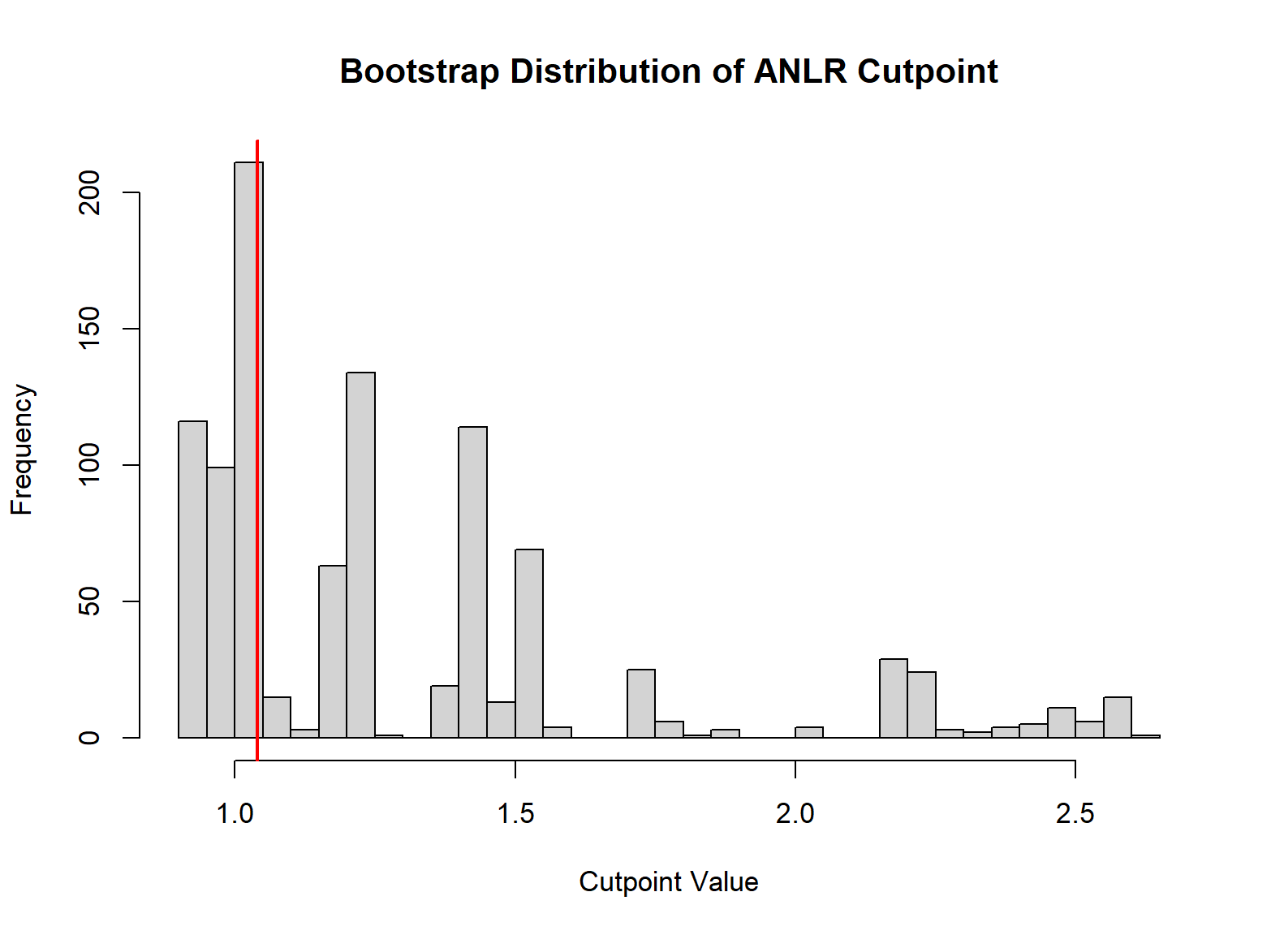


The Bootstrap analysis indicates that the distribution of the cutoff values of ANLR is concentrated and close to 1.04, suggesting that the result is stable. This stability reflects the minimal fluctuation of the cutoff values during repeated sampling, further validating the reliability of the model estimation. Moreover, the 95% confidence interval (CI) is ranging from 0.942 to 2.497, and the original cutoff value of 1.04 falls within this interval. This indicates that the cutoff estimate is statistically reliable. Therefore, considering both the Bootstrap distribution and the CI, the original cutoff estimate can be regarded as stable and robust.

**Figure S2.** The ROC curves of ANLR, NLR, and ALB on CVM and ACM in participants with advanced CKM syndrome


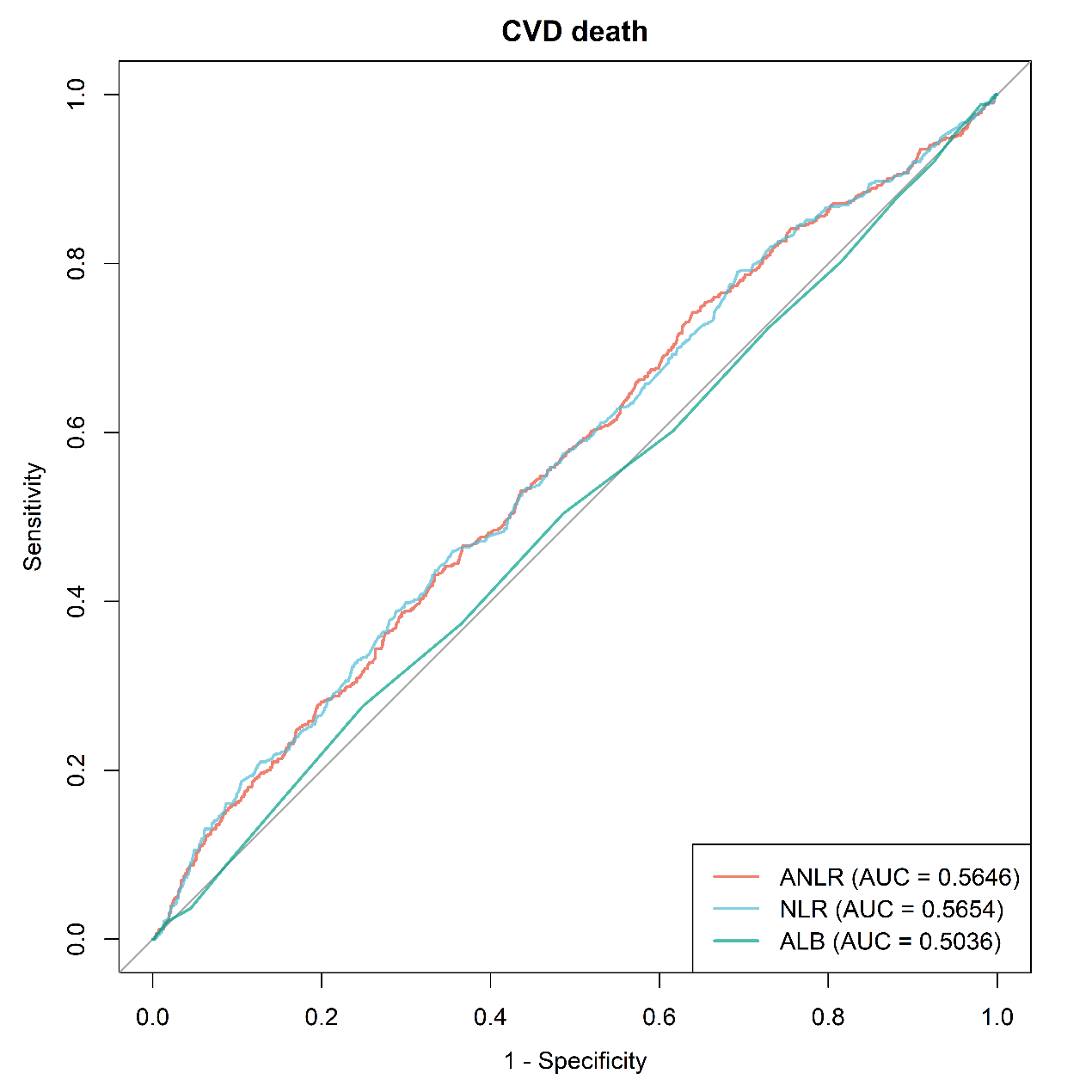

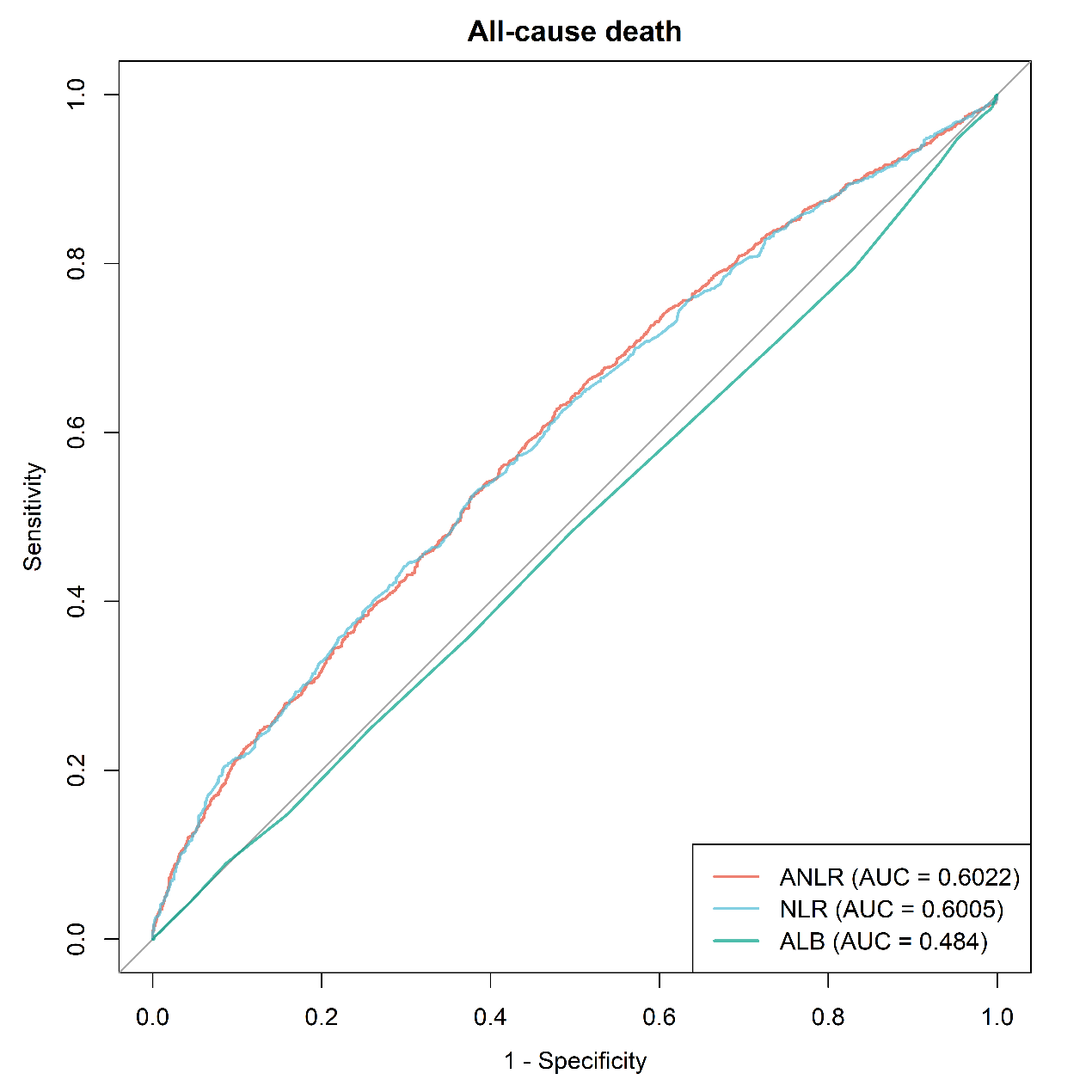

Supplement: Supplementary file 1 [file Table_1.DOCX]
